# Low ferroptosis score predicts chemotherapy responsiveness and immune‐activation in colorectal cancer

**DOI:** 10.1002/cam4.4956

**Published:** 2022-07-19

**Authors:** Yang Lv, Qing‐Yang Feng, Zhi‐Yuan Zhang, Peng Zheng, De‐xiang Zhu, Qi Lin, Si‐min Chen, Yi‐Hao Mao, Yu‐Qiu Xu, Mei‐ling Ji, Jian‐Min Xu, Guo‐dong He

**Affiliations:** ^1^ Department of General Surgery Zhongshan Hospital, Fudan University Shanghai China; ^2^ Colorectal Cancer Center Zhongshan Hospital, Fudan University Shanghai China; ^3^ Shanghai Engineering Research Center of Colorectal Cancer Minimally Invasive Surgery Shanghai China; ^4^ Department of Pathology Affiliated Hospital of Nanjing University of Chinese Medicine Nanjing Jiangsu China

**Keywords:** chemotherapy, colorectal cancer, ferroptosis, immune response, prognosis

## Abstract

**Background:**

Existing studies for ferroptosis and prognosis in colorectal cancer (CRC) were limited. In this study, we aim to investigate the prognostic role of ferroptosis markers in patients with CRC and exploration of its micro‐environmental distributions.

**Methods:**

Immunohistochemical staining was performed for CRC patients’ tissue microarray. Selection and prognostic validation of markers were based on mRNA data from the cancer genome atlas (TCGA) database. Gene Set Enrichment Analysis (GSEA) was performed to indicate relative immune landmarks and hallmarks. Ferroptosis and immune contexture were examined by CIBERSORT. Survival outcomes were analyzed by Kaplan‐Meier analysis and cox analysis.

**Results:**

A panel of 42 genes was selected. Through mRNA expression difference and prognosis analysis, GPX4, NOX1 and ACSL4 were selected as candidate markers. By IHC, increased GPX4, decreased NOX1 and decreased FACL4 indicate poor prognosis and worse clinical characteristics. Ferroptosis score based on GPX4, NOX1 and ACSL4 was constructed and validated with high C‐index. Low ferroptosis score can also demonstrate the better progression free survival and better adjuvant chemotherapy (ACT) responsiveness. Moreover, tumor with low ferroptosis score tend to be infiltrated with more CD4+ T cells, CD8+ T cells and less M1 macrophage. Finally, we found that IFN‐γ was potentially the central molecule at the crossroad between ferroptosis and onco‐immune response.

**Conclusion:**

Ferroptosis plays important role on CRC tumor progression, ACT response and prognosis. Ferroptosis contributes to immune‐supportive responses and IFN‐γ was the central molecule for this process.

## Graphical abstract

42 ferroptosis markers were reported, and GPX4, NOX1 and ACSL4 were candidate prognostic markers in colorectal cancer; Ferroptosis score based on expression of GPX4, NOX1 and ACSL4 could effectively reflect prognosis, tumor progression and adjuvant chemotherapy; Tumor with low ferroptosis score tend to be infiltrated with more CD4+ T cells, CD8+ T cells and less M1 macrophage; IFN‐γ was potentially the central molecule at the crossroad between ferroptosis and onco‐immune response.

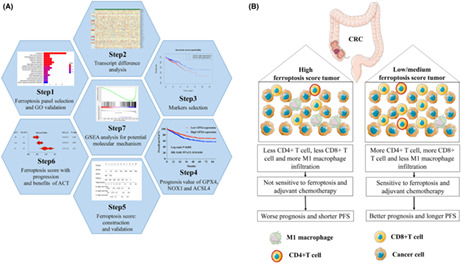

## 1 BACKGROUND

Colorectal cancer (CRC) is common around the world.^1^, ^2^ In China, CRC ranks the third most frequently diagnosed malignancy and the third leading cause of cancer‐associated mortality.^3^ Unfortunately, even after radical excision and systematic adjuvant chemotherapy (ACT), there were still 15%–25% CRC patients suffering from local and distant recurrence.^4^ Stratification for prognosis and treatment responsiveness of CRC is still warranted.

Ferroptosis is a new form of programmed cell death recognized by oxidative modification of membranes via an iron‐dependent mechanism.^5^ This iron‐dependent, non‐apoptotic form of cell death is characterized by lipid accumulation and can be induced by erastin,^6^ targeted therapy,^7^, ^8^ and chemotherapy.^9^, ^10^, ^11^, ^12^ Publications indicated potential role of ferroptosis on cancer translational medicine,^13^ including overcoming chemotherapy resistance^14^ and progression prevention.^15^ Analysis of sensitivity to ferroptosis stimuli across different cancers and cell lines were revealed and many related biomarkers were reported.^16^

However, clinical relations of ferroptosis and translational relevance on CRC were still lacking. Here, in this study, through candidate markers screen, three ferroptosis markers (GPX4, NOX1, and ACSL4) were selected. And we explored the clinical prognostic value of GPX4, NOX1, and ACSL4. Furthermore, a novel ferroptosis score based on GPX4, NOX1, and FACL4 were constructed and validated. Finally, immune micro‐environmental influences brought by ferroptosis were also explored.

## 2 METHODS

### 2.1 Patient eligibility and follow‐up principle

This study retrospectively enrolled consecutive 911 patients from Colorectal cancer center, Zhongshan Hospital, Fudan University (Shanghai, China) between 2008 and 2012. Of 911 patients, 528 were males. Postoperative ACT was administrated to patients according to the Chinese, NCCN CRC guidelines.^17^, ^18^, ^19^ This study was approved by the Ethical Committee of Zhongshan Hospital, Fudan University. Follow‐up principles were based on the Chinese guideline for colorectal cancer.^18^, ^20^

### 2.2 Ferroptosis markers selection

To determine a ferroptosis‐related markers list, an unbiased search for relevant articles was done on Pubmed (https://pubmed.ncbi.nlm.nih.gov/) for all full‐text articles pertaining to cancer and ferroptosis. Studies were identified using the term “cancer” OR “tumor” OR “neoplasm” AND “ferroptosis.” The date was from July 1, 1966 to July 1, 2020. Details were shown in Table S1 and Data S1.

### 2.3 TCGA data source and processing

Raw microarray datasets together with related clinical information were accessed from the database named Gene Expression Omnibus (GEO) (http://www.ncbi.nlm.nih.gov/geo/), and they were managed by Robust Multichip Average.^21^ Mapping of probes was performed based on the corresponding EntrezGeneID. The mean value of the probes was calculated when one EntrezGeneID got one more mapped probe. In this present research, we set the selection criterion as: (1) All datasets should be built through Affymetrix HG‐U133 plus 2.0 platform. (2) All datasets at least should have clinical information about AJCC stage, overall survival (OS) interval, and OS status. (3) The larger samples in individual dataset is preferred, the number of samples is at least 70 and above. (4) Datasets whose batch effects cannot be removed normally were deleted. Patients who were diagnosed as colon and rectal cancer were included in our study (Based on American Joint Committee on Cancer [AJCC] staging system). Combat was carried out o remove the batch effects among the different datasets and the process was carried out through R (www.r‐project.org/, Version 3.6.2) through package SVA. It contains 51 normal tissues and 647 tumor tissues. Significant up and down‐regulated genes were defined as fold change of at least 1.5× and adjusted *p*‐value ≤0.05. The results were visualized as a heat‐map plot using ggplot2 (RRID:SCR_014601) package.^22^ For further marker screening, prognostic value of each gene on CRC was determined.

### 2.4 Immunohistochemistry and intensity evaluation

Formalin‐fixed paraffin‐embedded surgical specimens were used for tissue microarray (TMA) construction and subsequent immunohistochemistry (IHC) study as described previously (Data S2).^23^, ^24^ Histological review was also conducted to avoid necrotic and hemorrhagic tumor regions. Immunoreactivity for GPX4, NOX1, and FACL4 in cancer cells was calculated as the product of two independent scores, the proportion of positive tumor cells in the tissues, and the average intensity of positive tumor cells in the tumor tissues.^25^ The CD4‐positive T cell, CD8‐positive T cell, and CD86‐positive M1 macrophage infiltration was recorded as the mean number of tryptase‐positive/HPF from three randomized fields.^24^ Expression was scored independently by two pathologists who were blinded to clinical‐pathological characteristics (Data S3). Besides, comparison between normal sections and TMA for IHC staining was demonstrated in Data S4. Cut‐off was determined as median score.

### 2.5 Antibodies

Antibodies composed of Rabbit anti‐human Glutathione Peroxidase 4 (GPX4, Abcam Cat#ab125066, RRID: AB_10973901), Rabbit anti‐human NOX1 (Abcam Cat# ab78016, RRID: AB_1566505), Rabbit anti‐human FACL4 (Abcam Cat# ab155282, RRID: AB_2714020), Rabbit anti‐CD4 (Abcam Cat# ab183685, RRID: AB_2686917), Rabbit anti‐CD8 (Abcam Cat# ab93278, RRID: AB_10563532), and Rabbit anti‐CD86 (Abcam Cat# ab119857, RRID: AB_10902800).

### 2.6 Construction of ferroptosis score on CRC prognosis

IHC score of GPX4, NOX1, and FACL4 was recorded on TMA. Patients were randomly divided into training set (455 patients) and validation set (456 patients). According to the ratio of 1:1, the training set was classified into the internal training group and the internal validation group. Multivariate Cox was performed to form the signature. Through Kaplan Meier survival analysis, risk score analysis, and ROC, the accuracy of the signature was confirmed. Risk score and some clinical features were included as risk factors and were further analyzed by univariate Cox analysis. Multivariate Cox analysis was utilized to analyze the risk factors which were considered significant after univariate Cox analysis. *p* < 0.05 was considered as statistically significant. Nomogram was constructed based on R studio (rms package). Construction of ferroptosis score (nomogram) and internal validation were performed based on multivariate cox analysis of OS from training set. To validate the statistical efficacy of ferroptosis score, data of validation set was also included for further analysis (external validation).

### 2.7 Analysis of immune infiltration

CIBERSORT was used to identify the characteristics of infiltrating immune cells. It can calculate cell numbers based on gene expression data from large pieces of tissue.^26^ Standardized data of gene expression was uploaded to the CIBERSORT web portal (http://cibersort.stanford.edu/). Finally, the abundance of 22 types of immune cells for each sample was calculated and the individual sample that was statistically significant was screened out. *p* < 0.05 was identified as statistically significant.

### 2.8 Gene set enrichment analysis

Gene set enrichment analysis (GSEA) was performed by the GSEA desktop application v.3.0 with 1000 permutations.^27^ Molecular Signatures Database (MSigDB) v6.0, was applied as a reference to determine pathways differentially enriched between low and high mRNA expression groups.^27^

### 2.9 Statistics

Statistical analyses were performed using the SPSS statistical package (22.0; SPSS; RRID:SCR_002865), R studio (R Project for Statistical Computing, RRID:SCR_001905), and prism 6 (GraphPad Prism, RRID:SCR_002798). NOX1, GPX4, and FACL4 expression between normal and cancer tissues was compared by paired Wilcoxon signed‐rank test. The correlations between continuous valuables were analyzed using Spearman rank correlation test and χ^2^ test. Time‐dependent cut‐off values were determined when positive likelihood ratio (PLR) was the largest one. Progression free survival (PFS) and Overall survival (OS) analyses were carried out using the Kaplan–Meier (KM) method and results were compared using a log‐rank test. A multivariable Cox proportional hazards model predicting OS was performed using backward stepwise selection. Risk factors were expressed as the hazard ratio [HR, 95% confidence interval (CI)]. Statistical significance was defined as *p*‐value less than 0.05.

## 3 RESULTS

### 3.1 Identification of prognostic ferroptosis markers in CRC

**FIGURE 1:**
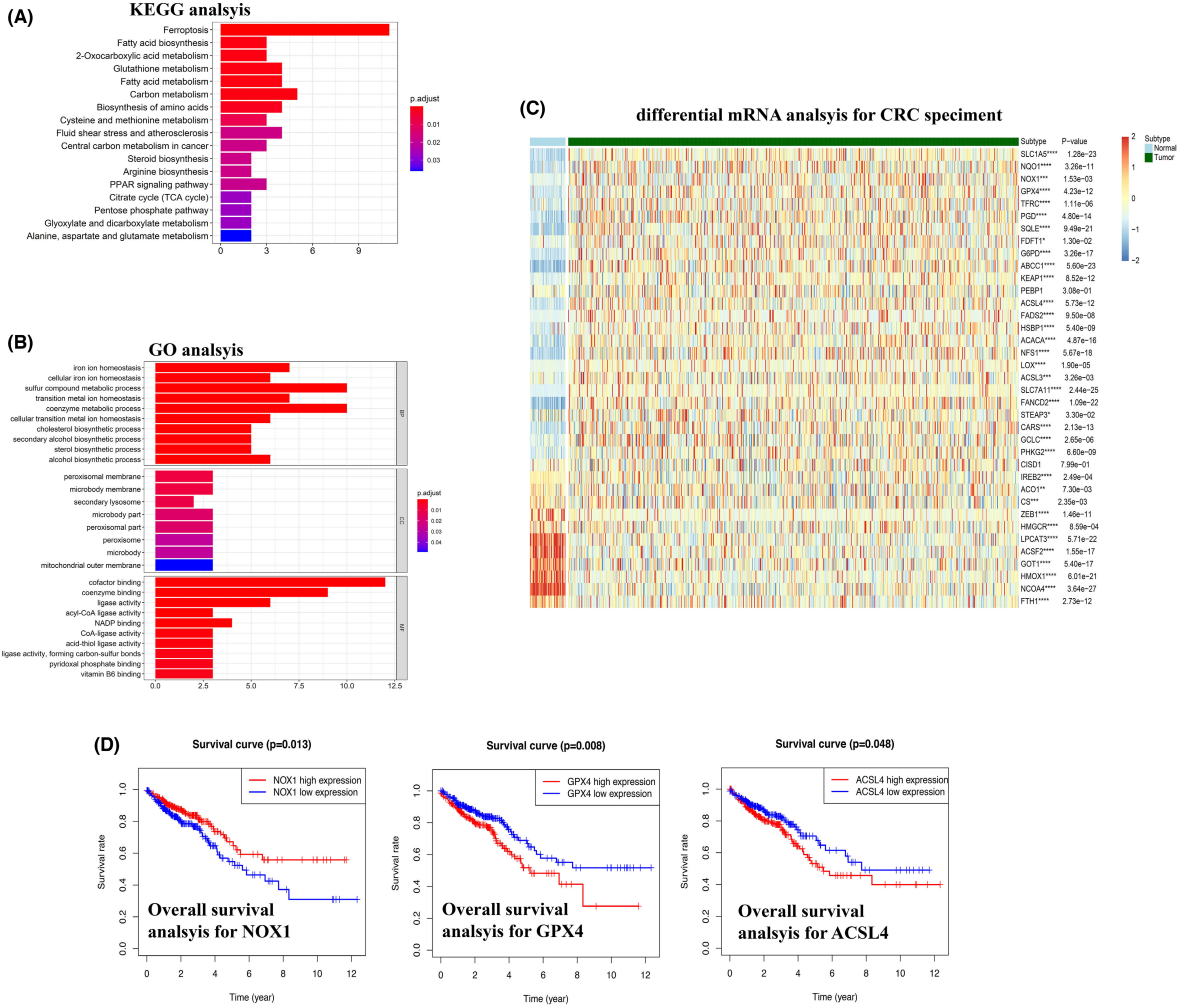
(A) KEGG analysis confirmed the key pathways for selected 42 genes and ferroptosis ranked the top pathway, the genes were selected from publication based on Pubmed database, and ferroptosis was involved from KEGG analysis; (B) GO analysis revealed potential cellular component (CC), molecular function (MF), and biological process (BP), the selected 42 genes were significantly correlated with iron ion homeostasis and peroxisomal membrane; (C) mRNA expression difference heatmap of 42 genes between CRC tumor and normal epithelium, the most significant 15 upregulated genes and 8 downregulated genes were selected for further survival analsysi; (D) Kaplan–Meier analysis of OS for GPX4, NOX1, and ACSL4, results demonstrated that higher expression of NOX1, low expression of GPX4, and low expression of ACSL4 separately demonstrated better prognosis in CRC patients from TCGA. CRC, colorectal cancer; GO, Gene ontology; KEGG, Kyoto Encyclopedia of Genes and Genomes; OS, overall survival

Ferroptosis‐related genes were selected according to publications^16^ and were shown in Table S1–S5. KEGG (Figure 1A), GO analysis (Figure 1B) and Protein–Protein interaction (PPI) network (Figure S1) of these genes were constructed to validate the biological relation with ferroptosis. Differential mRNA expression of these genes was constructed in Figure 1B and ranked by log fold changes (FC). Furthermore, KM analysis of the top 15 markers (*p* < 0.01) and the last 8 markers (*p* < 0.01) were performed to determine OS‐related role. In Figure 1C, low expression of NOX1 (*p* = 0.013), high expression of GPX4 (*p* = 0.008), and low expression of ACSL4 (*p* = 0.048) were separately regarded as risk factors for CRC patients' prognosis. Prognosis analysis of the other 16 genes were shown in Figure S2A (up‐regulated genes) and Figure S2B (down‐regulated genes).

### 3.2 Correlation between ferroptosis markers and clinical characteristics in CRC

**FIGURE 2:**
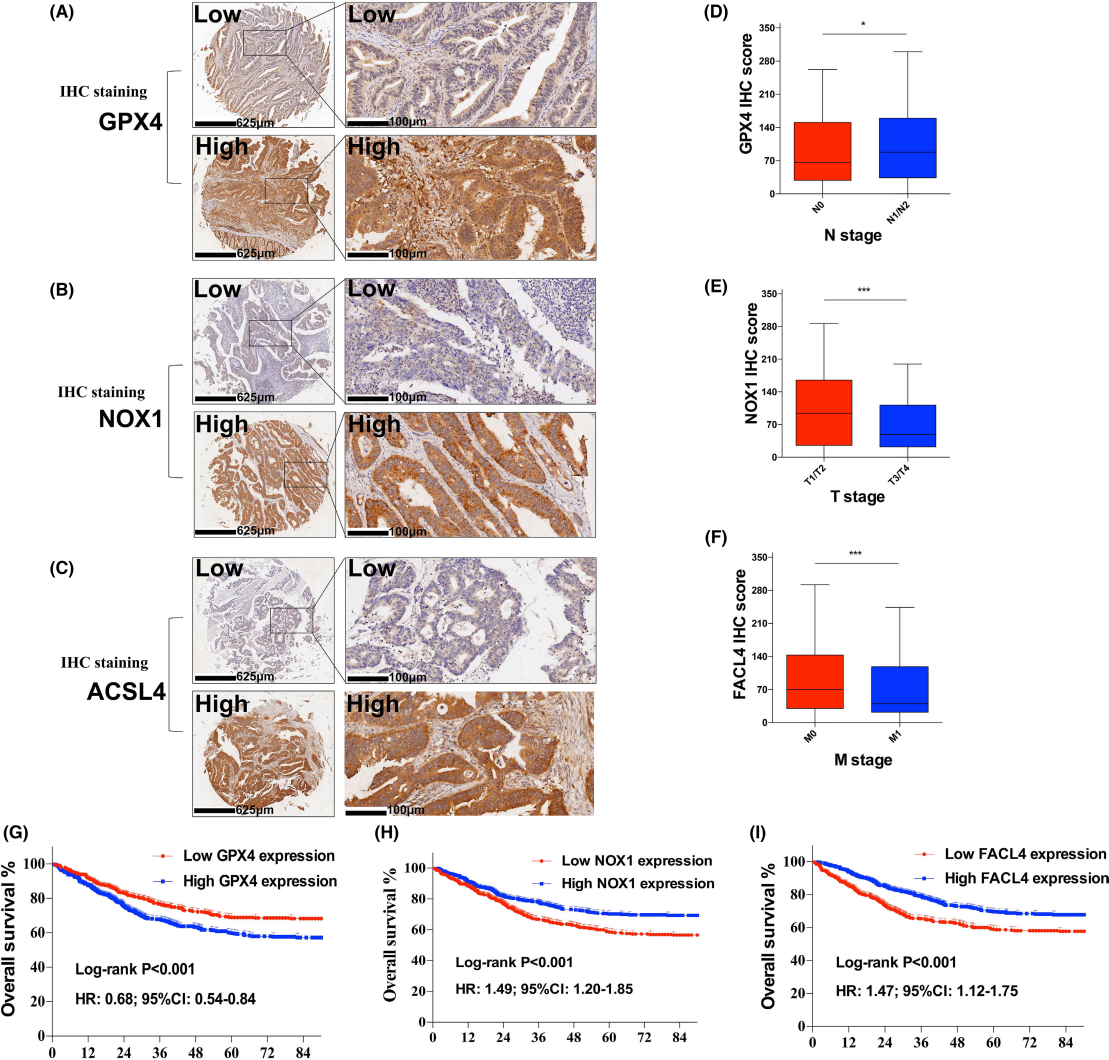
(A) Representative images of IHC staining for GPX4; (B) Representative images of IHC staining for NOX1; (C) Representative images of IHC staining for FACL4; (D) GPX4 IHC score between N0 stage and N1/N2 stage and higher expression of GPX4 demonstrated higher N stage; (E) NOX1 IHC score between T1/T2 stage and T3/T4 stage and lower expression of NOX1 demonstrated higher T stage; (F) ACSL4 IHC score between M0 stage and M1 stage and lower expression of ACSL4 demonstrated higher M stage; (G) Kaplan–Meier analysis of OS for GPX4 and higher expression of GPX4 demonstrated poorer prognosis; (H) Kaplan–Meier analysis of OS for NOX1 and lower expression of NOX1 demonstrated poorer prognosis; (I) Kaplan–Meier analysis of OS for FACL4 and lower expression of ACSL4 demonstrated poorer prognosis. CRC, colorectal cancer; IHC, immunohistochemistry; OS, overall survival

Protein expression of GPX4, NOX1, and FACL4 (ACSL4) were identified by IHC staining. Representative images were shown in Figure 2A–C). GPX4, NOX1, and FACL4 expression were higher than paired normal tissues (Figure S3A), which was further validated through Gene Expression Profiling Interactive Analysis (GEPIA) (Figure S3B).^28^

Before clinical analysis, biological role of GPX4, NOX1, and ACSL4 in CRC cells and ferroptosis was explored. As was shown in Figures S4 and S5, we generated GPX4 (Figure S5A), ACSL4 (Figure S4B), and NOX1 (Figure S5C)‐knockout HCT‐116 and HT‐29 cells model using SgRNA. By Soft‐agar analysis, three markers were also found biological importance on CRC cells (Figure S6A–C) and involved in CRC ferroptosis (Figure S6D).

Clinical correlation between three markers and clinical characteristics was shown in Table S2. Higher expression of GPX4, lower expression of NOX1 and FACL4 indicated larger primary tumor size (*p* = 0.001). Separately, higher expression of GPX4 were clinically correlated with higher lymph node metastasis (*p* = 0.029), lower NOX1 correlated with higher tumor invasion stage (*p* = 0.001), and lower FACL4 indicated more distant metastasis (*p* = 0.001), these were all demonstrated in Figure 2D–F.

### 3.3 Prognostic role of ferroptosis markers in CRC

KM survival analyses and Log‐rank tests were applied to evaluate prognostic merit in all CRC patients. As were shown in Figure 2G–I, low expression of GPX4 (*p* < 0.001; 95% CI: 0.54–0.84; HR:0.68), high expression of NOX1 (*p* < 0.001, 95% CI: 1.20–1.85; HR:1.49), and high expression of FACL4 (*p* < 0.001, 95% CI: 1.12–1.75; HR:1.47) demonstrated better prognosis in patients with CRC. Univariate and Multivariate analysis were shown in Table S3, expression of GPX4 (*p* = 0.014, 95% CI: 0.58–0.94; HR: 0.74), NOX1 (*p* = 0.026, 95% CI: 1.03–1.67; HR:1.31), and FACL4 (*p* = 0.015, 95% CI: 1.21–1.66; HR:1.34) were independent factors for OS in CRC. Event‐based ROC curves for OS were constructed based on GPX4, NOX1, and FACL4, respectively. AUC were separately 0.57 for GPX4 (Figure S7A), 0.53 for NOX1 (Figure S7B), and 0.56 for FACL4 (Figure S7C). Furthermore, time dependent ROCs were constructed to determine the prognostic role of ferroptosis‐related markers, Figure S7 indicated survival dependent AUCs of GPX4 (Figure S7D), NOX1 (Figure S7E), and FACL4 (Figure S7F) for OS.

### 3.4 Subgroup analysis of GPX4, NOX1, and FACL4 for OS in CRC

**FIGURE 3:**
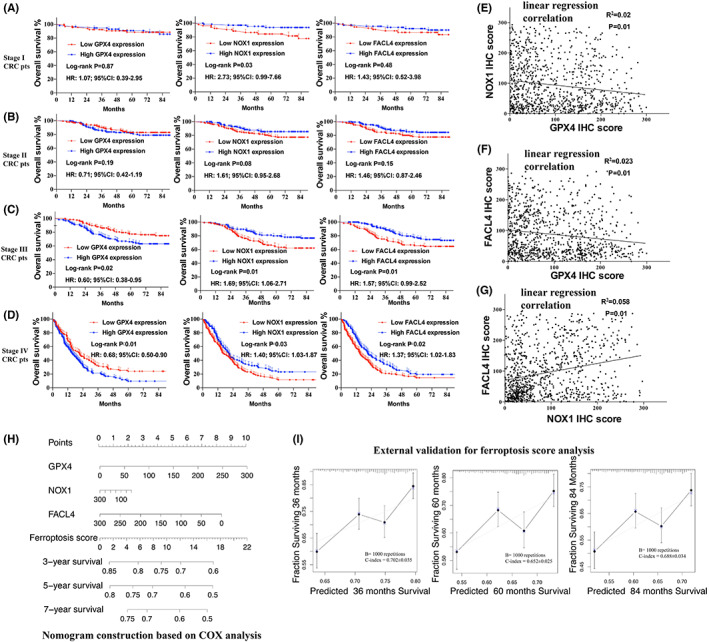
(A) Kaplan–Meier analysis of GPX4, NOX1, and FACL4 expression on OS in Stage I CRC patients, GPX4, and FACL4 expression demonstrated no significance for Stage I CRC patients survival; (B) Kaplan–Meier analysis of GPX4, NOX1, and FACL4 expression on OS in Stage II CRC patients, GPX4 and FACL4 expression demonstrated no significance for Stage II CRC patients survival; (C) Kaplan–Meier analysis of GPX4, NOX1, and FACL4 expression on OS in Stage III CRC patients, all GPX4, NOX1, and FACL4 expression demonstrated significance for Stage III CRC patients survival; (D) Kaplan–Meier analysis of GPX4, NOX1, and FACL4 expression on OS in Stage IV CRC patients, all GPX4, NOX1, and FACL4 expression demonstrated significance for Stage IV CRC patients survival; (E) IHC score correlation between GPX4 and NOX1; (F) IHC score correlation between GPX4 and FACL4; (G) IHC score correlation between FACL4 and NOX1; (H) Construction of ferroptosis score based on multivariable Cox analysis; (I) Validation of ferroptosis score on 3, 5 and 7 years' survival rate based on validation cohort data. CRC, colorectal cancer; IHC, immunohistochemistry; OS, overall survival

In subgroup analysis, patients were divided into four groups according to AJCC stage: Stage I, Stage II, Stage III, and Stage IV. In Stage I CRC (Figure 3A), expression of GPX4 (*p* = 0.87, HR: 1.07, 95%CI: 0.39–2.95) and FACL4 (*p* = 0.48, HR: 1.43, 95%CI: 0.52–3.98) have no prognostic role for OS, while patients with low level of NOX1 had worse survival outcomes (*p* = 0.03, HR: 1.07, 95%CI: 0.39–2.95). In Stage II CRC (Figure 3B), expression of three individual marker demonstrated no significant prognostic role (*p* > 0.05). And in Stage III and Stage IV CRC, GPX4, NOX1, and FACL4 were regarded as significant prognosis‐related factors (all *p* < 0.05, Figure 3C,D).

### 3.5 Ferroptosis score for prognosis in CRC: development and validation

IHC score relations among GPX4, NOX1, and FACL4were determined in Figure 3E–G (*p* < 0.05). To assess the comprehensive ferroptosis status, all 911 patients were divided randomly into 2 cohorts (training and validation cohort). Baseline characteristics were shown in Table S4 and there was no difference between training and validation cohort. Clinical model incorporating GPX4, NOX1, and FACL4 was constructed based on characteristics of training cohort. Figure 3H conferred nomogram for CRC prognosis and calibration curves were presented high agreement between predicted survival and actual survival in both training cohort (Figure S8) and validation cohort (Figure 3I). Furthermore, C‐index combining ferroptosis score with TNM staging yield higher accuracy than TNM alone in both sets (Table S5). Subgroup analysis for different TNM stage were shown in Figure S9.

### 3.6 Ferroptosis score as a predictive parameter for tumor progression in CRC

**FIGURE 4:**
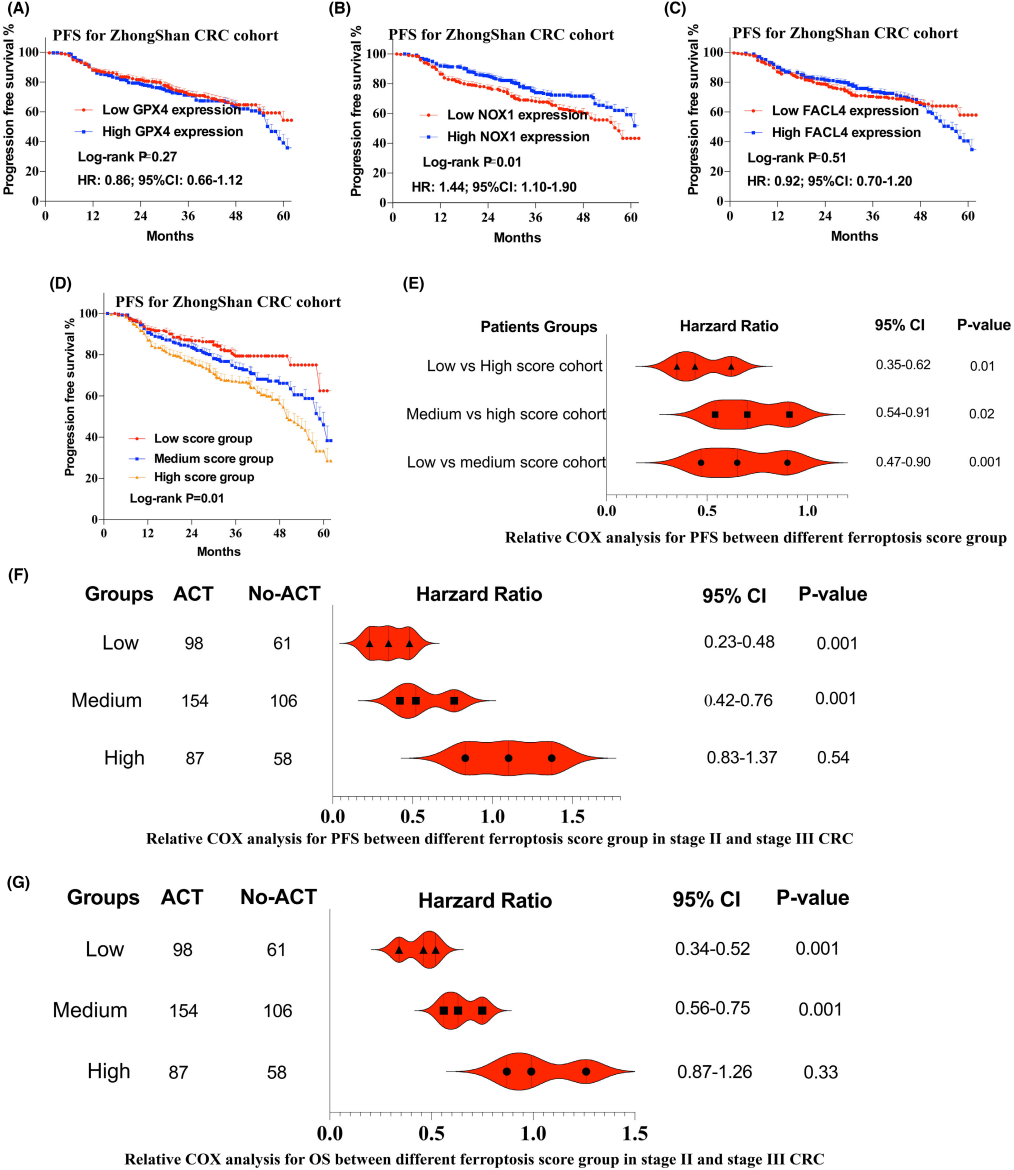
Kaplan–Meier analysis of GPX4 (A), NOX1 (B) and FACL4 (C) expression on PFS from Zhongshan CRC cohort patients, GPX4 and FACL4 solely demonstrated no statistical significance on CRC patients' PFS; (D) Kaplan–Meier analysis of ferroptosis score on PFS stratification, low ferroptosis score patients had significant longer PFS interval than medium score group and high score group (*p* = 0.01); (E) Cox proportional hazards regression analysis for comparisons of PFS in different Ferroptosis risk groups; (F) Cox proportional hazards regression PFS analysis for the difference of responsiveness to ACT within different risk groups; (G) Cox proportional hazards regression OS analysis for the difference of responsiveness to ACT within different risk groups. ACT, adjuvant chemotherapy; CRC, colorectal cancer; OS, overall survival; PFS, progression free survival

KM method was used for PFS analysis. For all patients, individual GPX4 and FACL4 expression has no role on DFS stratification (*p* > 0.05), while expression of NOX1 had statistical correlation with PFS (*p* < 0.05). Details were shown in Figure 4A–C (IHC findings) and Figure S10 (TCGA findings). Ferroptosis score was further divided into three groups: High score group (14–22 scores), medium score group (7–14 scores), and low score group (0–7 scores). Low‐score group patients demonstrated favorable PFS, while high‐score group patients demonstrated worst PFS (Figure 4D). Furthermore, we performed Cox regression analysis to assess the relationship among different groups. As was shown in Figure 4E, ferroptosis score could be regarded as an independent prognostic factor to predict recurrence for CRC patients.

### 3.7 Ferroptosis score with ACT in stage II and III CRC

Furthermore, we sought to discover whether different risk groups indicated distinct responsiveness to ACT in CRC patients. For Stage IV CRC patients, not all patients underwent radical resection for metastases and treatment regimens often incorporated targeted therapies. To be more precise, we focused on ACT benefits in Stage II and III patients. Results indicated ferroptosis score could be used to stratify patients into different risk subgroups, low and median ferroptosis score patients benefited more from ACT and had better PFS (Figure 4F) and OS (Figure 4G) (*p* < 0.001). In contrast, high score patients had inferior therapeutic responsiveness to ACT. Details for PFS and OS between ACT and no‐ACT cohorts were shown in Figure S11.

### 3.8 Elements of ferroptosis score system shape immune contexture in CRC

**FIGURE 5:**
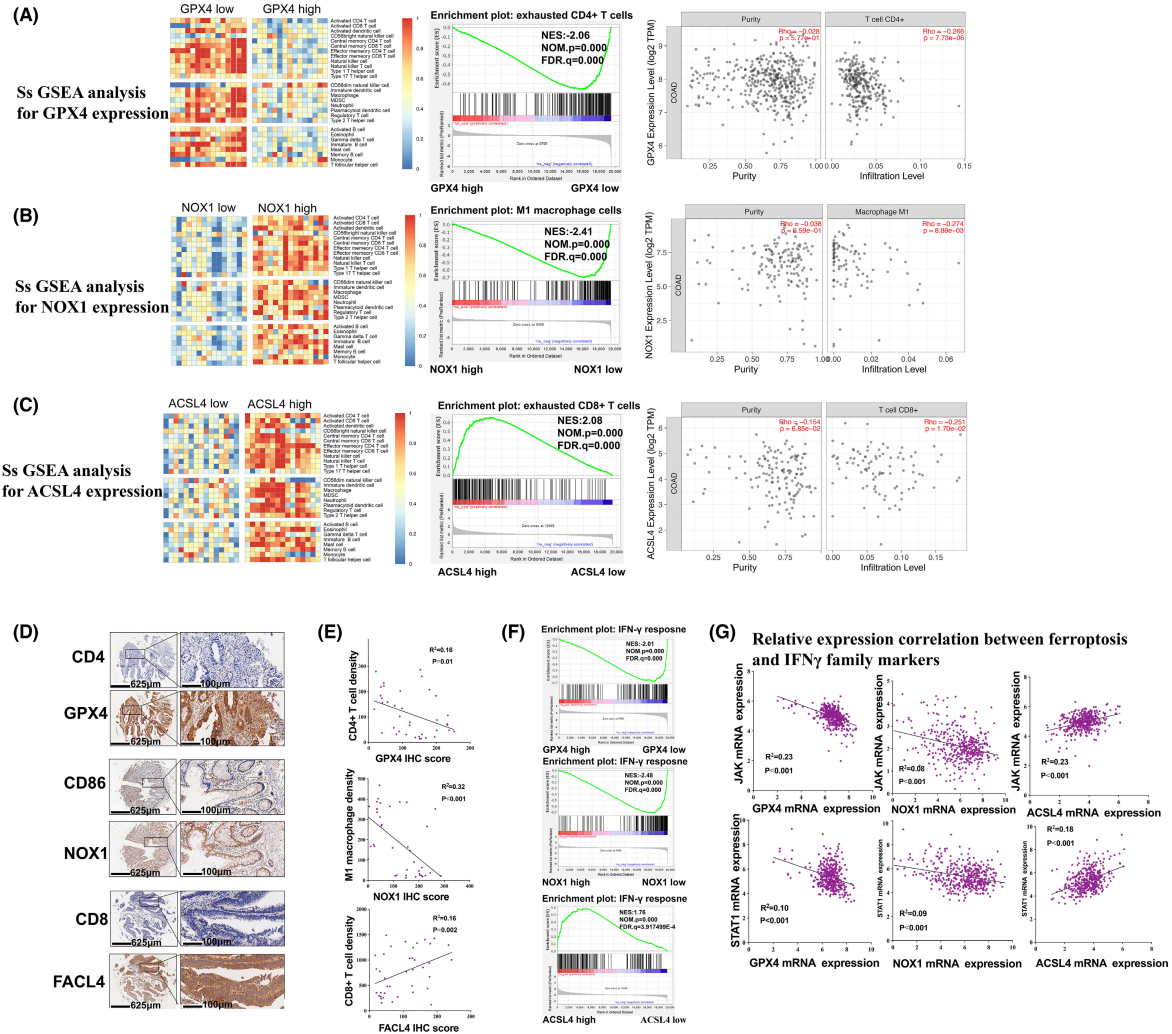
(A) GSEA analysis for GPX4 expression and CD4+ T cell infiltration; (B) GSEA analysis for NOX1 expression and M1 macrophage infiltration; (C) GSEA analysis for ACSL4 expression and CD8+ T cell infiltration; (D) Representative images of double staining of CD4 and GPX4 (top) or CD86 and NOX1 (medium) or CD8 and FACL4 (bottom) on TMA and Zhongshan CRC cohort TMA validated the significant correlation for GSEA analysis; (E) scatter diagram between ferroptosis markers and immune infiltration; (F) GSEA analysis for hallmarks of cancer on GPX4, NOX1, and ACSL4 mRNA expression; (G) scatter diagram between ferroptosis markers and downstream of IFN‐γ (JAK and Stat1). GSEA, gene set enrichment analysis; TMA, tissue microarray

By using GSEA to compare the mRNA expression profile between low and high GPX4, NOX1, and ACSL4, respectively. SsGSEA analysis demonstrated changes of immune contexture brought by the ferroptosis markers (Figure 5A–C, left part). Multiple immune‐related pathways were found, including CD4+ T cells **(**Figure 5A, middle part), CD8+ T cells (Figure 5B, middle part) and M1 macrophage (Figure 5C, middle part). As was shown in Figure 5A, CD4+ T cells pathway were significantly enriched in low GPX4 groups with the Normalized Enrichment Score (NES) of −2.06 (*p* = 0.000), these results were validated by CIBERSORT analysis which indicated GPX4 expression was significantly negatively correlated with CD4+ T cell infiltration **(**Figure 5A, right part). Also, expression correlations between M1 macrophage and NOX1 (NSE = −2.41, *p* = 0.000), CD8+ T cell infiltration and ACSL4 (NSE = 2.08, *p* = 0.000) were indicated through GSEA and CIBERSORT analysis (Figure 5B,C).

IHC of CD4, CD8, and CD86 were further performed for validation. Representative images of double staining were shown in Figure 5D. Analysis demonstrated that GPX4 was negatively correlated with CD4+ T cell infiltration (*R* ^2^ = 0.16, *p* = 0.01), NOX1 expression was negatively correlated with M1 macrophage infiltration (*R* ^2^ = 0.32, *p* < 0.001) and FACL4 expression was positively correlated with CD8+ T cell infiltration (*R* ^2^ = 0.16, *p* = 0.002). Details were shown in Figure 5E.

### 3.9 IFN‐γ were potentially involved in tumor ferroptosis and indicated better prognosis in CRC

By GSEA for Hallmarks, interferon‐γ (IFN‐γ) response were all significantly enriched for all three markers (low GPX4, low NOX1, and high ACSL4) (Figure 5F). The NES were separately −2.01, −2.48, and 1.76 for GPX4, NOX1, and ACSL4. GPX4 and NOX1 were negatively correlated with IFN‐γ (*p* < 0.001), while ACSL4 were positively correlated with IFN‐γ (*p* < 0.001).

To further validate the results, JAK expression and stat1 expression was evaluated. As was demonstrated in Figure 5G, GPX mRNA expression was negatively correlated with JAK (*R* ^2^ = 0.23, *p* < 0.001) and Stat1 (*R* ^2^ = 0.10, *p* < 0.001), NOX1 mRNA expression was negatively correlated with JAK (*R* ^2^ = 0.08, *p* < 0.001) and stat1 (*R* ^2^ = 0.09, *p* < 0.001) and ACSL4 mRNA expression was positively correlated with JAK (*R* ^2^ = 0.23, *p* < 0.001) and stat1 (*R* ^2^ = 0.18, *p* < 0.001). All these results were consistent with GSEA findings, indicated ferroptosis score were highly correlated with anti‐tumor immunity, and IFN‐γ may be the key regulator at the crossroad between ferroptosis and anti‐tumor immunity. Furthermore, based GEPIA cohort analysis, high expression of IFNG were statistically demonstrated with longer duration of DFS (*p* = 0.018) (Figure S12).

## 4 DISCUSSION

**FIGURE 6:**
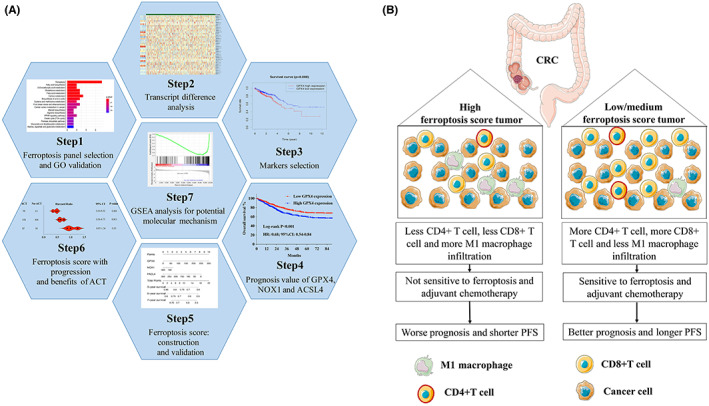
(A) Graphical summary of this study and Schematics depicting the materials and methods used in the research; (B) the prognostic role of ferroptosis and the underlying immune‐activation functions on CRC. CRC, colorectal cancer

Since the discovery of ferroptosis, targeting ferroptosis has been regarded as a novel anti‐cancer strategy,^29^ compelling evidence indicated that compounds like erastin and sorafenib could induce tumor ferroptosis.^30^ Besides, potential molecular mechanisms involved in ferroptosis were observed in many experimental cancer models,^31^, ^32^, ^33^ including inactivation of GPX4, up‐regulation of FACL4. However, given the promising opportunities and challenges, in CRC, relevance between ferroptosis and clinical characteristics was still little known. Figure 6A demonstrated diagram for this study and Figure 6B conferred to the prognostic role of ferroptosis score and the potential mechanisms.

Tumor ferroptosis is a complex process. This process can be modulated through many pathways and communicated by other microenvironment cells.^34^ Given the close relation between ferroptosis and cell metabolism, truly CRC ferroptosis status may not be reflected by single gene. Through transcript difference analysis and prognosis screen, we selected a small panel including GPX4, NOX1, and ACSL4 for further study.

Realizing proteins as the “executioners of life” that determine phenotype, IHC assays for GPX4, NOX1, and FACL4(ACSL4) were performed on TMA and recorded by independent pathologist. Results were reported to be same robustness as classic tumor sections.^35^ In our study, prognostic value of protein level was highly consistent with mRNA level expression. Low expression of GPX4 indicated better survival, high expression of NOX1 and FACL4 separately demonstrated better survival in CRC. Then, high GPX4 expression was significantly correlated with lymph‐node metastasis (*p* = 0.029), tumor with low NOX1 expression tend to be higher pathological T stage (*p* = 0.001) and low expression of FACL4 was statistically correlated with higher M stage, besides, all the three markers expression were clinically correlated with primary tumor size (all *p* = 0.001).

We further determine AUC of OS for each marker. AUC of GPX4, NOX1, and FACL4 were separately merely 0.57, 0.53, and 0.56. Time‐dependent AUC was also not more than 0.65. These results indicated again that single ferroptosis marker was not enough to reflect the real ferroptosis status. We wonder whether combining GPX4, NOX1, and FACL4 could complementarily present tumor ferroptosis status in CRC. In the next step, by randomly dividing our patients into two groups, a nomogram (ferroptosis score) based on COX multivariable analysis was first construed and validated. Besides, incorporating ferroptosis to classic TNM stage could effectively improve the prognosis prediction power on CRC. In the next step, ferroptosis score could also stratify patients into different tumor progression groups (PFS), analysis of ACT responsiveness demonstrated the same result. Compared to high ferroptosis score cohort, CRC patients' tumor with low/medium ferroptosis score would be easier to benefit from ACT.

Down‐regulation of GPX4,^36^, ^37^ up‐regulation of ACSL4^38^ and up‐regulation of NOX1^39^ facilitate ferroptosis sensitivity through different pathways. Potential mechanisms have been reported. However, so far, immune‐modulation role of ferroptosis sensitive tumor cells was not revealed. Dying cells, mostly in the context of ferroptosis sensitivity, communicate with immune cells by a set of signals.^40^ This facilitates immune cells to locate ferroptotic cells in the tissue and mediate the movement of immune cells within tissues. We first reported microenvironment influences brought by ferroptosis sensitive tumor cells. Based on GSEA and CIBERSORT analysis, tumor cells with low GPX4 tend to be infiltrated by higher proportion of CD4+ T cell, expression of NOX1 was negatively correlated with M1 macrophage infiltration and ACSL4 was positively close to CD8+ T cell infiltration. To further validate these findings, double IHC staining of CD4 and GPX4, CD86 and NOX1, CD8 and FACL4 was performed and results confirmed the relations. Recently reports demonstrated that IFN‐γ produced by tumor‐infiltrating T cells contributed to tumor ferroptosis.^41^, ^42^ In our study, through cancer hallmarks analysis, IFN‐γ was regarded as a central gene at the cross‐roads between ferroptosis and immune responses in CRC. Furthermore, downstream factors expression (JAK and stat1) was validated and consistent with results of GSEA. This could be reasonable that higher infiltration of CD4+ T and CD8+ T cells resulted in higher expression of IFN‐γ, which facilitates tumor ferroptosis and then patients' prognosis. On the other hand, reduced IFN‐γ could decrease the process of M0 Macrophages into M1 polarization,^43^ which was consistent to our results.

There are still some limitations. First, the candidate list with regard to ferroptosis‐related markers was based on previous publications and there may be more precise and specific markers for CRC which was still uncovered yet. Second, to conduct a real‐world study, we enrolled consecutive patients to continue the study cohort. There were inevitable imbalances at the baseline, especially in the application of ACT. This imbalance could interfere with the results. Third, for nomogram construction, two cohorts of patients came from the same medical center, thus lacking of external validation. Finally, the central role of IFN‐γ was determined and validated through bioinformatics analysis. More detailed experimental works of molecular mechanisms are still required in the future.

In summary, based on current publications, our study showed GPX4, NOX1, and ACSL4 were significant prognostic ferroptosis‐related markers in CRC. Ferroptosis markers was closely related with clinical characteristics; ferroptosis score based on GPX4, NOX1, and FACL4 can effectively reflect CRC prognosis, tumor progression and ACT responsiveness with high C‐index. Furthermore, tumor with low ferroptosis score may be infiltrated with more CD4+ T cells, CD8+ T cells, and less M1 macrophage. In this process, IFN‐γ may be potentially the central role at the crossroad between ferroptosis and onco‐immune response.

## AUTHORS' CONTRIBUTIONS

Yang Lv, QingYang Feng, ZhiYuan Zhang, and Peng Zheng analyzed and interpreted the patient data. De‐Xiang Zhu collected the clinical data, and Yang Lv was a major contributor in writing the manuscript. Pro JianMin Xu and Pro GuoDong He contributed to the design of the work and were the corresponding authors in this manuscript. SiMin Chen, YuQiu Xu, YiHao Mao, MeiLing Ji, and Qi Lin provided the research background and perspective views. Pro GuoDong He and Pro JianMin Xu were the corresponding authors and approved the final version of this manuscript to be published.

## FUNDING INFORMATION

This work was supported by National Natural Science Foundation of China (Grant No. 81602040, 81903067, and 81402341), Clinical science and technology innovation project of Shanghai (SHDC12016104), Shanghai Science and Technology Committee Project (17411951300, 18140903200, 19140900703, 20DZ2201400), and Youth fund of Zhongshan Hospital (2019ZSQN28). The funding bodies had no role in the design of the study and collection, analysis, and interpretation of data and in the writing of the manuscript.

## CONFLICT OF INTEREST

The authors declare no conflicts of interest for the publication of this manuscript.

## ETHICS APPROVAL AND CONSENT TO PARTICIPATE

Written informed consent was obtained by all the patients. The study protocol followed the ethical guidelines of the Declaration of Helsinki and was approved by the Ethical Committee of Zhongshan Hospital of Fudan University. The ethics approval ID was B2017‐166R.

## CONSENT FOR PUBLICATION

We have obtained consent to publish from the participant to report individual patient data.

## Supporting information


Table S1–S5
Figure S1–S12
Data S1–S4

## Data Availability

The datasets used and/or analysed during the current study are available from the corresponding authors on reasonable request.
