# Supplementary material for: Low ferroptosis score predicts chemotherapy responsiveness and immune‐activation in colorectal cancer
Source: Cancer Med. 2022 Jul 19;12(2):2033–45. doi: 10.1002/cam4.4956 (PMC9883409; doi:10.1002/cam4.4956)
Supplement: Supplementary file 1 — Table S1–S5 Figure S1–S12 Data S1–S4 [file CAM4-12-2033-s001.docx]

**Supplementary content for**

**Low ferroptosis score predicts chemotherapy responsiveness and immune-activation in colorectal cancer**

**Author:** *Yang Lv, Qing-Yang Feng, Zhi-Yuan Zhang, Peng Zheng, De-xiang Zhu,* *Si-min Chen, Yi-Hao Mao, Yu-Qiu Xu, Mei-ling Ji, Jian-Min Xu,* *Guo-dong He*

**Correspondence to:** Guo-Dong He, [angelhgd@163.com](mailto:angelhgd@163.com); and Jian-Min Xu, [xujmin@aliyun.com](mailto:xujmin@aliyun.com); Department of General Surgery, Zhongshan Hospital, Fudan University.

**This file includes:**

1. Supplementary Materials and Methods

2. Tables S1 to S5

3. Figures. S1 to S12

4. Supplementary information 1 to 4

**Supplementary information content for**

1. **Supplementary methods and materials**
2. **Supplementary tables**

**Supplementary table S1** Ferroptosis related genes according to publications

**Supplementary table S2** Relationship between significant markers and clinical characteristics in CRC patients

**Supplementary table S3** Univariate and multivariate analysis for OS among 911 CRC patients

**Supplementary table S4** Baseline clinicopathological characteristics of training and validation cohorts

**Supplementary table S5** Comparison of predictive accuracies of prognostic models

1. **Supplementary figures**

**Supplementary figure S1** PPI network of ferroptosis panel constructed based on TCGA database. **Abbreviation:** PPI, protein-to-protein interaction; TCGA, the cancer genome atlas.

**Supplementary figure S2 (A)** Kaplan-meier analysis of up-regulated genes on CRC OS; **(B)** Kaplan-meier analysis of down-regulated genes on OS. **Abbreviation:** OS, overall survival; CRC, colorectal cancer.

**Supplementary figure S3 (A)** IHC score between cancer tissue and normal epithelium based on CRC TMA; **(B)** mRNA expression between cancer tissue and normal epithelium in CRC. **Abbreviation:** IHC, immunohistochemistry; CRC, colorectal cancer; TMA, tissue microarray.

**Supplementary figure S4** Confirmation of Crispr-knouk-out targeting GPX4 **(A)**, ACSL4 **(B)** and NOX1 **(C)**.

**Supplementary figure S5** Construction of GPX4 **(A)**, ACSL4 **(B)** and NOX1 **(C)** knock out model in CRC cell lines. **Abbreviation:** CRC, colorectal cancer

**Supplementary figure S6**. Knock out of GPX4 **(A)**, ACSL4 **(B)** and NOX1 **(C)** on CRC cell viability; **(D)** GPX4, ACSL4 and NOX1 was involved in CRC ferroptosis. **Abbreviation:** CRC, colorectal cancer

**Supplementary figure S7** ROC curves and AUC value for GPX4 **(A)**, NOX1 **(B)** and FACL4 **(C)** on CRC OS and Time-dependent AUC for OS on GPX4 **(D)**, NOX1 **(E)** and FACL4 **(F)**. **Abbreviation:** ROC, receiver operating characteristic; OS, overall survival.

**Supplementary figure S8** Internal validation of ferroptosis score on 3 years, 5 years and 7 years’ survival based on training cohort data.

**Supplementary figure S9** Kaplan-meier analysis of ferroptosis score on OS stratification and Cox proportional hazards regression analysis for comparisons of OS in stage I **(A)**, stage II **(B)**, stage III **(C)** and stage IV **(D)** CRC; **Abbreviation:** OS, overall survival; CRC, colorectal cancer.

**Supplementary figure S10** Kaplan-meier analysis of GPX4 **(A)**, NOX1 **(B)** and ACSL4 **(C)** on PFS based on TCGA analysis. **Abbreviation:** PFS, progression free survival; TCGA, the cancer genome atlas.

**Supplementary figure S11** In low ferroptosis score cohort, stage II and III patients received ACT had better duration of PFS **(A)** and OS **(D)** than no ACT patients; In medium ferroptosis score, stage II and III patients received ACT had better duration of PFS **(B)** and OS **(E)** than no ACT patients; In high ferroptosis score, patients received ACT had the similar duration of PFS **(C)** and OS **(G)** than no ACT patients. **Abbreviation:** CRC, colorectal cancer; PFS, progression free survival; ACT, adjuvant chemotherapy; OS, overall survival.

**Supplementary figure S12** Kaplan-meier analysis of IFN-γ on CRC RFS. **Abbreviation:** CRC, colorectal cancer; RFS, relapse free survival.

1. **Supplementary information**

**Supplementary information 1** Exploration of Ferroptosis-related gene in pubmed

**Supplementary information 2** Tissue microarray construction, immunohistochemical staining

**Supplementary information 3** Evaluation of the stability and repeatability of the detection method for ferroptosis-related genes

**Supplementary information 4** Comparison between normal sections and tissue microarray for immunohistochemical staining

**Supplementary materials and methods**

1. **Plasmid constructs and lentivirus production**

The empty vector (EV) was purchased from GeneCopoeiaTM (#EX-Z2866-Lv105). Short guide RNAs (sgRNAs) that target human GPX4, ACSL4 and NOX1 were designed using the Optimized CRISPR Design web tool (http://crispr.mit.edu/). SgRandom control was constructed by cloning a sgRNA with a random sequence into the lentiCRISPRv2 vector. Lentivirus particles were generated by co-transfecting these lentiviral constructs with helper virus packaging plasmids pCMVΔR8.9 and pHCMV-VSV-G into HEK293T cells using Lipofectamine 3000 (Invitrogen# L3000015). Lentiviruses were harvested after 24, 48 and 72 h, and frozen at –80°C in aliquots at appropriate amounts for infection.

1. **CRISPR knockout**

Short guide RNAs (sgRNAs) targeting human GPX4, ACSL4 and NOX1were designed on the basis of the Opimized CRISPR Design web tool (http://crispr.mit.edu/) and cloned into lentiCRISPRv2 vector (Addgene, plasmid #52961) individually. HCT116 and HT29 cells were infected for 16 hours in the supernatant containing 8μg/ml polybrene (Sigma; #107689), and then treated with puromycin (Millipore; #540411) one day after infection. Knock-out was confirmed by sanger sequence data in **Figure S4**.

1. **Real-time quantitative RT-PCR and quantitative PCR**

Cell line tissues were homogenized in TRIzol Reagent (Invitrogen; #15596026), followed by total RNA isolation using the standard protocol. The RNA was further reverse-transcribed into cDNA using the TransScript First-Strand cDNA Synthesis SuperMix (TransGen; #AT301). Genomic DNA was isolated using QIAamp DNA Mini Kit (QIAGEN; #51306). qPCR was performed for target-gene-expression analysis or exon detection of GPX4, ACSL4 and NOX1 using the iQTM SYBR Green Supermix (Bio-Rad; #170-8882). Samples were run in triplicate with non-reverse transcriptase or non-template control. Amplification accuracy was verified by melting curve analysis. Relative mRNA expression was normalized to GAPDH expression as an internal amplification control. The primers for GPX4 were 5′- GAGGCAAGACCGAAGTAAACTAC-3′ and 5′- CCGAACTGGTTACACGGGAA-3′; ACSL4 were 5’-CGGTTCCTTTTTGCGAGCTT-3’ and 5’-AAAGTACGCAAATGTCCTCTTTT-3’; NOX1 were 5’-GTTTTACCGCTCCCAGCAGAA-3′ and 5’-GGATGCCATTCCAGGAGAGAG-3′; GAPDH were 5’-AGGGCTGCTTTTAACTCTGGT-3′ and 5’-CCCCACTTGATTTTGGAGGGA-3′.

1. **Western Blot analysis**

Whole cell lysates from cell lines were prepared using lysis buffer (1% NP-40, 50 mM Tris-HCl pH 8.0, 100 mM sodium fluoride, 30 mM sodium pyrophosphate, 2 mM sodium molybdate, 5mM EDTA, 2 mM sodium orthovanadate) containing or not containing protease inhibitors. The lysates were then rocked overnight at 4℃ and cleared by centrifugation at 14,000rpm for 30min at 4℃. The lysate protein concentrations were determined using a Quick StartTM Bradford 1× Dye Reagent (Bio-Rad; #5000205). Electrophoresis and western blotting were performed using standard techniques. The hybridization signals were detected by chemiluminescence (Immobilon Western, Millipore Corporation, MA) and captured using an Amersham Imager 600 imagers (GE Healthcare; #29083461). Cells were grown until 80% confluence, washed with PBS and harvested. Primary antibodies were GPX4 (abcam#125066), NOX1 (abcam#131088), FACL4 (abcam#155282).

1. **Soft agar assay**

Six-well plates were first layered with 0.6% bottom agar (Noble agar, BD difco #214220) containing RPMI 1640 medium with 10% FBS and penicillin/streptomycin. CRC cell line (2 X 10^4^ /per well), were seeded in 0.4% top agar containing 10% FBS and penicillin/streptomycin. Cells were allowed to grow for 3-5 weeks and then stained with 1 ml of 1 mg/ml methyl thiazol tetrazolium (Sigma–Aldrich #M5655) for 3 h. Colonies were counted manually, and the clone size was measured by Image-J software (National Institutes of Health). All assays were performed in triplicate wells, with the entire study replicated at least once.

**Supplementary tables**

**Table S1:** **ferroptosis related gene according to publications**

| **Iron Metabolism** | NCOA4^1,2^, ACO1^2^, FTH1^3^, STEAP3^4^, FANCD2^4,5^, NFS1^6,7^, TFRC^8,9^, PHKG2^10^, IREB2^11^, HSBP1^12^, HMOX1^13,14^, CISD1/mitoNEET^15^, |
| --- | --- |
| **Lipid Metabolism** | ACSF2^11^, CS(citrate synthase )^11^, LPCAT3^16^ , ACSL4^16,17^, ACSL3^18^, ACACA^16^, GPX4^19^, AKR1C^20^, LOX^10,21^, PEBP1^22^, ZEB1^23^, SQS/FDFT1^24,25^, SQLE^25^, HMGCR^24^, FADS2^26^ |
| **Energy Metabolism** | SLC1A5^8^, GLS2^8^, GOT1^8^, G6PD^2,11^, PGD^11^ |
| **Oxidant Metabolism** | NRF2^3,27^, KEAP1^28^, HMOX1^29^, NQO1^3^, SLC7A11^11^, GCLC^19^, CARS^30^, CBS^30^, NOX1^11^, ABCC1/MRP1^2^ |

**References:**

1. Hou W, Xie Y, Song X, et al. Autophagy promotes ferroptosis by degradation of ferritin. Autophagy. 2016;12(8):1425-1428.

2. Gao M, Monian P, Pan Q, Zhang W, Xiang J, Jiang X. Ferroptosis is an autophagic cell death process. Cell Res. 2016;26(9):1021-1032.

3. Sun X, Ou Z, Chen R, et al. Activation of the p62-Keap1-NRF2 pathway protects against ferroptosis in hepatocellular carcinoma cells. Hepatology. 2016;63(1):173-184.

4. Song X, Xie Y, Kang R, et al. FANCD2 protects against bone marrow injury from ferroptosis. Biochem Biophys Res Commun. 2016;480(3):443-449.

5. Li C, Zhang Y, Liu J, Kang R, Klionsky DJ, Tang D. Mitochondrial DNA stress triggers autophagy-dependent ferroptotic death. Autophagy. 2020:1-13.

6. Alvarez SW, Possemato R. Leveraging the iron-starvation response to promote ferroptosis. Oncotarget. 2018;9(13):10830-10831.

7. Alvarez SW, Sviderskiy VO, Terzi EM, et al. NFS1 undergoes positive selection in lung tumours and protects cells from ferroptosis. Nature. 2017;551(7682):639-643.

8. Gao M, Monian P, Quadri N, Ramasamy R, Jiang X. Glutaminolysis and Transferrin Regulate Ferroptosis. Mol Cell. 2015;59(2):298-308.

9. Yang WS, Stockwell BR. Synthetic lethal screening identifies compounds activating iron-dependent, nonapoptotic cell death in oncogenic-RAS-harboring cancer cells. Chem Biol. 2008;15(3):234-245.

10. Yang WS, Kim KJ, Gaschler MM, Patel M, Shchepinov MS, Stockwell BR. Peroxidation of polyunsaturated fatty acids by lipoxygenases drives ferroptosis. Proc Natl Acad Sci U S A. 2016;113(34):E4966-4975.

11. Dixon SJ, Lemberg KM, Lamprecht MR, et al. Ferroptosis: an iron-dependent form of nonapoptotic cell death. Cell. 2012;149(5):1060-1072.

12. Sun X, Ou Z, Xie M, et al. HSPB1 as a novel regulator of ferroptotic cancer cell death. Oncogene. 2015;34(45):5617-5625.

13. Hassannia B, Wiernicki B, Ingold I, et al. Nano-targeted induction of dual ferroptotic mechanisms eradicates high-risk neuroblastoma. J Clin Invest. 2018;128(8):3341-3355.

14. Chang LC, Chiang SK, Chen SE, Yu YL, Chou RH, Chang WC. Heme oxygenase-1 mediates BAY 11-7085 induced ferroptosis. Cancer Lett. 2018;416:124-137.

15. Yuan H, Li X, Zhang X, Kang R, Tang D. CISD1 inhibits ferroptosis by protection against mitochondrial lipid peroxidation. Biochem Biophys Res Commun. 2016;478(2):838-844.

16. Dixon SJ, Winter GE, Musavi LS, et al. Human Haploid Cell Genetics Reveals Roles for Lipid Metabolism Genes in Nonapoptotic Cell Death. ACS Chem Biol. 2015;10(7):1604-1609.

17. Vanden Berghe T, Hassannia B, Vandenabeele P. An outline of necrosome triggers. Cell Mol Life Sci. 2016;73(11-12):2137-2152.

18. Magtanong L, Ko PJ, To M, et al. Exogenous Monounsaturated Fatty Acids Promote a Ferroptosis-Resistant Cell State. Cell Chem Biol. 2019;26(3):420-432 e429.

19. Yang WS, SriRamaratnam R, Welsch ME, et al. Regulation of ferroptotic cancer cell death by GPX4. Cell. 2014;156(1-2):317-331.

20. Dixon SJ, Patel DN, Welsch M, et al. Pharmacological inhibition of cystine-glutamate exchange induces endoplasmic reticulum stress and ferroptosis. Elife. 2014;3:e02523.

21. Seiler A, Schneider M, Forster H, et al. Glutathione peroxidase 4 senses and translates oxidative stress into 12/15-lipoxygenase dependent- and AIF-mediated cell death. Cell Metab. 2008;8(3):237-248.

22. Wenzel SE, Tyurina YY, Zhao J, et al. PEBP1 Wardens Ferroptosis by Enabling Lipoxygenase Generation of Lipid Death Signals. Cell. 2017;171(3):628-641 e626.

23. Viswanathan VS, Ryan MJ, Dhruv HD, et al. Dependency of a therapy-resistant state of cancer cells on a lipid peroxidase pathway. Nature. 2017;547(7664):453-457.

24. Shimada K, Skouta R, Kaplan A, et al. Global survey of cell death mechanisms reveals metabolic regulation of ferroptosis. Nat Chem Biol. 2016;12(7):497-503.

25. Garcia-Bermudez J, Baudrier L, Bayraktar EC, et al. Squalene accumulation in cholesterol auxotrophic lymphomas prevents oxidative cell death. Nature. 2019;567(7746):118-122.

26. Zhang Y, Tan H, Daniels JD, et al. Imidazole Ketone Erastin Induces Ferroptosis and Slows Tumor Growth in a Mouse Lymphoma Model. Cell Chem Biol. 2019;26(5):623-633 e629.

27. Roh JL, Kim EH, Jang H, Shin D. Nrf2 inhibition reverses the resistance of cisplatin-resistant head and neck cancer cells to artesunate-induced ferroptosis. Redox Biol. 2017;11:254-262.

28. Fan Z, Wirth AK, Chen D, et al. Nrf2-Keap1 pathway promotes cell proliferation and diminishes ferroptosis. Oncogenesis. 2017;6(8):e371.

29. Adedoyin O, Boddu R, Traylor A, et al. Heme oxygenase-1 mitigates ferroptosis in renal proximal tubule cells. Am J Physiol Renal Physiol. 2018;314(5):F702-F714.

30. Hayano M, Yang WS, Corn CK, Pagano NC, Stockwell BR. Loss of cysteinyl-tRNA synthetase (CARS) induces the transsulfuration pathway and inhibits ferroptosis induced by cystine deprivation. Cell Death Differ. 2016;23(2):270-278.

**Table S2. Relationship between significant markers and clinical characteristics in CRC patients**

|  | **Ferroptosis markers** | | | | | | | | |
| --- | --- | --- | --- | --- | --- | --- | --- | --- | --- |
|  | **GPX4** | | | **NOX1** | | | **FACL4** | | |
| **Factors** | Low (%) | High (%) | *P* | Low (%) | High (%) | *P* | Low (%) | High (%) | *P* |
| **All patients** | 463 | 448 |  | 459 | 452 |  | 450 | 461 |  |
| **Age (years)** |  |  | 0.088 |  |  | 0.228 |  |  | 0.358 |
| ≤60 | 187 (40.4) | 206 (46.0) |  | 189 (41.2) | 204 (45.1) |  | 201 (44.7) | 192 (41.6) |  |
| >60 | 276 (59.6) | 242 (54.0) |  | 270 (58.8) | 96 (54.9) |  | 249 (55.3) | 269 (58.4) |  |
| **Gender** |  |  | 0.753 |  |  | 0.500 |  |  | 0.609 |
| Male | 266 (57.5) | 262 (58.5) |  | 261 (56.9) | 267 (59.1) |  | 257 (57.1) | 271 (58.8) |  |
| Female | 197 (42.5) | 186 (41.5) |  | 198 (43.1) | 185 (40.9) |  | 193 (42.9) | 190 (41.2) |  |
| **CEA (ng/ml)** |  |  | 0.423 |  |  | 0.353 |  |  | 0.142 |
| ≤ 5 | 252 (54.4) | 222 (49.6) |  | 232 (50.5) | 242  (53.5) |  | 220 (48.9) | 254 (55.1) |  |
| > 5 | 200 (43.2) | 215 (48.0) |  | 213 (46.4) | 202 (44.7) |  | 217 (48.2) | 198 (43.0) |  |
| Unknown | 11  (2.4) | 11 (2.5) |  | 14 (3.1) | 8 (1.8) |  | 13 (2.9) | 9 (2.0) |  |
| **Tumor location** |  |  | 0.192 |  |  | 0.393 |  |  | 0.637 |
| Right-sided colon | 121 (26.1) | 133 (29.7) |  | 136 (29.6) | 118 (26.1) |  | 129 (28.7) | 125 (27.1) |  |
| Left-sided colon | 112 (24.2) | 122 (27.2) |  | 120 (26.1) | 114 (25.2) |  | 117 (26.0) | 117 (25.4) |  |
| Rectum | 230 (49.7) | 193 (43.1) |  | 203 (44.2) | 220 (48.7) |  | 204 (45.3) | 219 (47.5) |  |
| **Tumor size** |  |  | **0.001** |  |  | **0.001** |  |  | **0.001** |
| ≤4.0cm | 256 (55.3) | 129 (28.8) |  | 123 (26.8) | 262 (58.0) |  | 263 (59.4) | 122 (26.5) |  |
| >4.0cm | 207 (44.7) | 319 (71.2) |  | 336 (73.2) | 190 (42.0) |  | 180 (40.6) | 339 (73.5) |  |
| **Primary histological type** |  |  | 0.427 |  |  | 0.468 |  |  | 0.949 |
| Non-mucinous | 387 (83.6) | 383 (85.5) |  | 384 (83.7) | 386 (85.4) |  | 380 (84.4) | 390 (84.6) |  |
| Mucinous | 76 (16.4) | 65 (14.5) |  | 75 (16.3) | 66 (14.6) |  | 70 (15.6) | 71 (15.4) |  |
| **Primary differentiation** |  |  | **0.009** |  |  | 0.425 |  |  | 0.868 |
| Well/moderate | 349 (73.8) | 295 (65.8) |  | 330 (71.1) | 314 (68.7) |  | 317 (69.7) | 327 (70.2) |  |
| Poor/anaplastic | 124 (26.2) | 153 (34.2) |  | 134 (28.9) | 143 (31.3) |  | 138 (30.3) | 139 (29.8) |  |
| **T stage** |  |  | 0.960 |  |  | **0.001** |  |  | 0.565 |
| T1/T2 | 224 (48.4) | 216 (48.2) |  | 180 (39.2) | 260 (57.5) |  | 213 (47.3) | 227 (49.2) |  |
| T3/T4 | 239 (51.6) | 232 (51.8) |  | 279 (60.8) | 192 (41.8) |  | 237 (52.7) | 234 (50.8) |  |
| **N stage** |  |  | **0.029** |  |  | 0.937 |  |  | 0.096 |
| N0 | 269 (58.1) | 228 (50.9) |  | 251 (54.7) | 246 (54.4) |  | 233 (51.8) | 264 (57.3) |  |
| N1/N2 | 194 (41.9) | 220 (49.1) |  | 208 (45.3) | 206 (45.6) |  | 217 (48.2) | 197 (42.7) |  |
| **Vascular/nerve invasion** |  |  | 0.891 |  |  | **0.001** |  |  | **0.021** |
| No | 386 (83.4) | 375 (83.7) |  | 356 (77.6) | 405 (89.6) |  | 363 (80.7) | 398 (86.3) |  |
| Yes | 77 (16.6) | 73 (16.3) |  | 103 (22.4) | 47 (10.4) |  | 87 (19.3) | 63 (13.7) |  |
| **M stage** |  |  | 0.057 |  |  | 0.136 |  |  | **0.001** |
| M0 | 363 (78.4) | 327 (73.0) |  | 338 (73.6) | 352 (77.9) |  | 313 (69.9) | 377 (81.8) |  |
| M1 | 100 (21.6) | 121 (27.0) |  | 121 (26.4) | 100 (22.1) |  | 137 (30.4) | 84 (18.2) |  |

Abbreviations: T, tumor invasion depth; N, lymph node involvement; M, metastasis.

| **Characteristics** | **Univariate analysis** | | | **Multivariate analysis** | | |
| --- | --- | --- | --- | --- | --- | --- |
|  | **Pvalue** | **HR** | **95%CI** | **Pvalue** | **HR** | **95%CI** |
| **Gender** | 0.078 |  |  |  |  |  |
| Male |  | 1(Reference) | 1(Reference) |  |  |  |
| Female |  | 1.223 | 0.843-1.526 |  |  |  |
| **Age** | 0.945 |  |  |  |  |  |
| ≥60 |  | 1(Reference) | 1(Reference) |  |  |  |
| <60 |  | 1.008 | 0.809-1.255 |  |  |  |
| **Primary site** | 0.085 |  |  |  |  |  |
| Left sided colon |  | 1(Reference) | 1(Reference) |  |  |  |
| Right sided colon |  | 0.173 | 0.024-1.246 |  |  |  |
| Rectum |  | 0.122 | 0.024-1.267 |  |  |  |
| **Tumor size** | 0.035 |  |  | 0.163 |  |  |
| ≤4.0cm |  | 1(Reference) | 1(Reference) |  | 1(Reference) | 1(Reference) |
| >4.0cm |  | 1.235 | 1.012-1.561 |  | 1.185 | 0.934-1.505 |
| **Differentiation** | 0.001 |  |  | 0.023 |  |  |
| Well/moderate |  | 1(Reference) | 1(Reference) |  | 1(Reference) | 1(Reference) |
| Poor/anaplastic |  | 1.654 | 1.321-2.069 |  | 1.331 | 1.040-1.703 |
| **T stage** | 0.001 |  |  | 0.197 |  |  |
| T1/T2 |  | 1(Reference) | 1(Reference) |  | 1(Reference) | 1(Reference) |
| T3/T4 |  | 2.813 | 1.884-4.201 |  | 1.341 | 0.859-2.904 |
| **N stage** | 0.001 |  |  | 0.001 |  |  |
| N0 |  | 1(Reference) | 1(Reference) |  | 1(Reference) | 1(Reference) |
| N1/N2 |  | 2.886 | 2.297-3.626 |  | 1.724 | 1.315-2.259 |
| **M stage** | 0.001 |  |  | 0.001 |  |  |
| M0 |  | 1(Reference) | 1(Reference) |  | 1(Reference) | 1(Reference) |
| M1 |  | 8.197 | 6.579-10.309 |  | 2.631 | 2.315-2.994 |
| **Vascular/nerve invasion** | 0.078 |  |  |  |  |  |
| No |  | 1(Reference) | 1(Reference) |  |  |  |
| Yes |  | 0.625 | 0.446-1.372 |  |  |  |
| **GPX4** | 0.001 |  |  | 0.014 |  |  |
| High expression |  | 1(Reference) | 1(Reference) |  | 1(Reference) | 1(Reference) |
| Low expression |  | 0.678 | 0.542-0.837 |  | 0.740 | 0.581-0.941 |
| **NOX1** | 0.001 |  |  | 0.026 |  |  |
| High expression |  | 1(Reference) | 1(Reference) |  | 1(Reference) | 1(Reference) |
| Low expression |  | 1.491 | 1.203-1.845 |  | 1.311 | 1.033-1.665 |
| **FACL4** | 0.001 |  |  | 0.015 |  |  |
| High expression |  | 1(Reference) | 1(Reference) |  | 1(Reference) | 1(Reference) |
| Low expression |  | 1.47 | 1.12-1.75 |  | 1.336 | 1.213-1.656 |

**Table S3: Univariate and multivariate analysis for OS among 911 CRC patients**

**Table S4. Baseline clinicopathological characteristics of training and validation cohorts**

|  | **Training set** | | **Validation set** | | P-value |
| --- | --- | --- | --- | --- | --- |
| **Factors** | No. | % | No. | % |  |
| **All patients** | 455 | 100.0 | 456 | 100 |  |
| **Age (years)** |  |  |  |  | 0.660 |
| ≤ 60 | 193 | 42.4 | 200 | 43.9 |  |
| > 60 | 262 | 57.6 | 256 | 56.1 |  |
| **Gender** |  |  |  |  | 0.969 |
| Male | 264 | 58.0 | 264 | 58.0 |  |
| Female | 191 | 42.0 | 192 | 42.0 |  |
| **CEA (ng/ml)** |  |  |  |  | 0.097 |
| ≤ 5 | 238 | 52.3 | 236 | 51.8 |  |
| > 5 | 211 | 46.4 | 204 | 44.7 |  |
| Unknown | 6 | 1.3 | 16 | 3.5 |  |
| **Tumor location** |  |  |  |  | 0.906 |
| Right-sided colon | 128 | 28.1 | 126 | 27.6 |  |
| Left-sided colon | 121 | 26.6 | 122 | 26.8 |  |
| Rectum | 206 | 45.3 | 217 | 47.6 |  |
| **Tumor size** |  |  |  |  | 0.399 |
| ≤ 4.0cm | 186 | 40.9 | 199 | 43.6 |  |
| > 4.0cm | 269 | 59.1 | 257 | 56.4 |  |
| **Primary histological type** |  |  |  |  | 0.938 |
| Non-mucinous | 385 | 84.6 | 385 | 84.4 |  |
| Mucinous | 70 | 15.4 | 71 | 15.6 |  |
| **Primary differentiation** |  |  |  |  | 0.477 |
| Well/moderate | 323 | 71.8 | 321 | 69.6 |  |
| Poor/ anaplastic | 127 | 28.2 | 140 | 30.4 |  |
| **T stage** |  |  |  |  | 0.574 |
| T1/T2 | 224 | 49.2 | 216 | 47.4 |  |
| T3/T4 | 231 | 50.8 | 240 | 52.6 |  |
| **N stage** |  |  |  |  | 0.442 |
| N0 | 254 | 55.8 | 243 | 53.3 |  |
| N1/N2 | 201 | 44.2 | 213 | 46.7 |  |
| **Nerve/Vascular invasion** |  |  |  |  | 0.988 |
| No | 375 | 82.4 | 376 | 82.5 |  |
| Yes | 80 | 17.6 | 80 | 17.5 |  |
| **M stage** |  |  |  |  | 0.406 |
| M0 | 350 | 76.9 | 340 | 74.6 |  |
| M1 | 105 | 23.1 | 116 | 25.4 |  |

**Table S5. Comparison of predictive accuracies of prognostic models**

|  | **Training cohort** | | | **Validation cohort** | | |
| --- | --- | --- | --- | --- | --- | --- |
| **Factor** | **C-index** | **95%CI** | **P-value** | **C-index** | **95%CI** | **P-value** |
| Ferroptosis score with TNM | 0.721 | 0.682-0.751 | Reference | 0.715 | 0.693-0.742 | Reference |
| Ferroptosis score | 0.692 | 0.654-0.739 | <0.001 | 0.681 | 0.665-0.716 | <0.001 |
| GPX4 | 0.570 | 0.537-0.616 | <0.001 | 0.565 | 0.535-0.621 | <0.001 |
| NOX1 | 0.526 | 0.492-0.573 | <0.001 | 0.531 | 0.502-0.585 | <0.001 |
| FACL4 | 0.562 | 0.528-0.614 | <0.001 | 0.554 | 0.532-0.608 | <0.001 |
| Pathological T stage | 0.622 | 0.565-0.668 | <0.001 | 0.632 | 0.598-0.674 | <0.001 |
| Lymph nodes metastasis | 0.610 | 0.545-0.654 | <0.001 | 0.601 | 0.573-0.645 | <0.001 |
| Distant metastasis | 0.682 | 0.643-0.726 | <0.001 | 0.665 | 0.638-0.728 | <0.001 |
| TNM | 0.701 | 0.635-0.745 | 0.035 | 0.697 | 0.675-0.721 | 0.029 |

Abbreviations: C-index, Harrell’s Concordance index; TNM, tumor node metastasis; CI, confidence interval.

**Supplementary figures**

Supplementary Figure S1
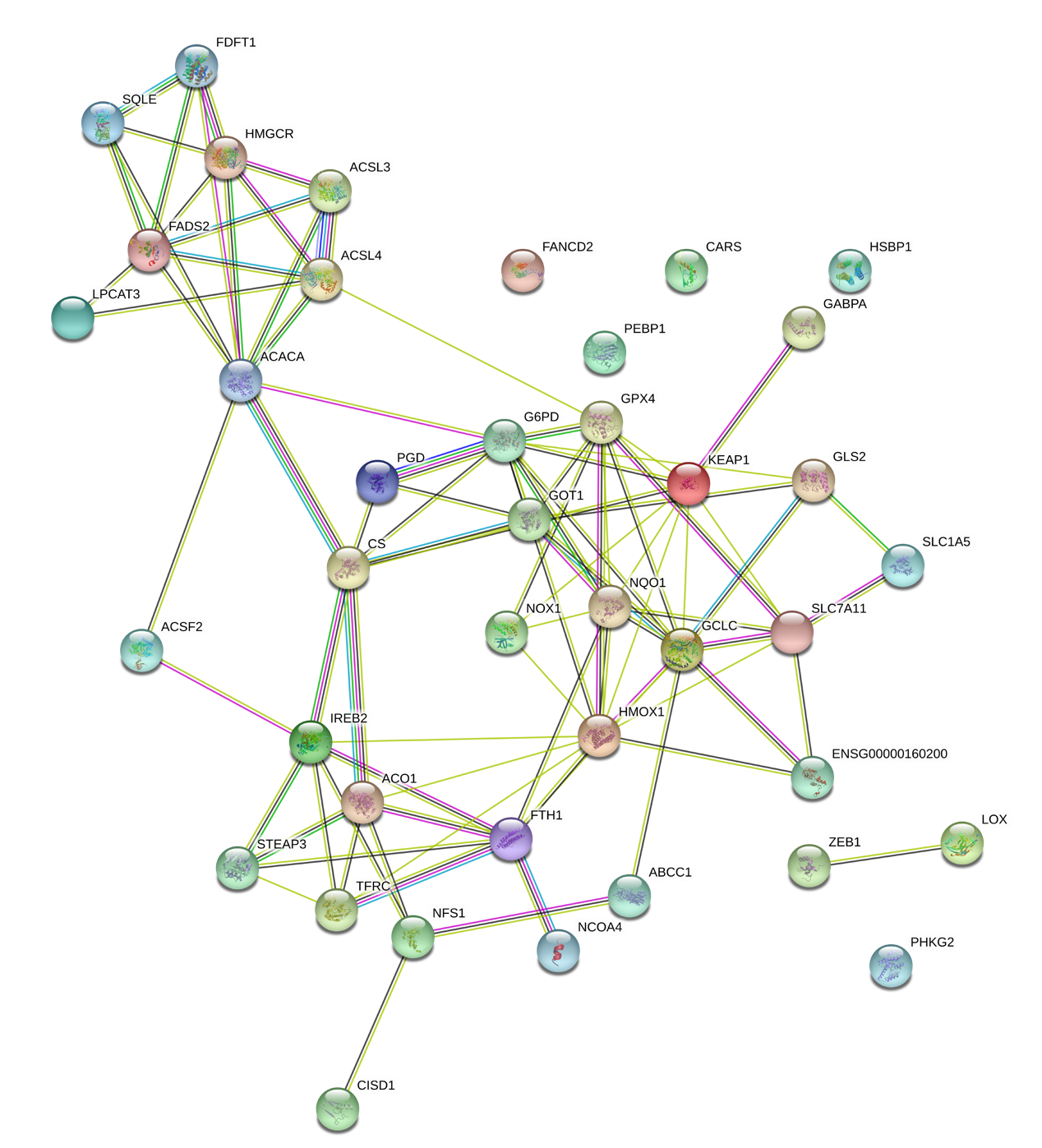


Supplementary Figure S2

Supplementary Figure S3

Supplementary Figure S4


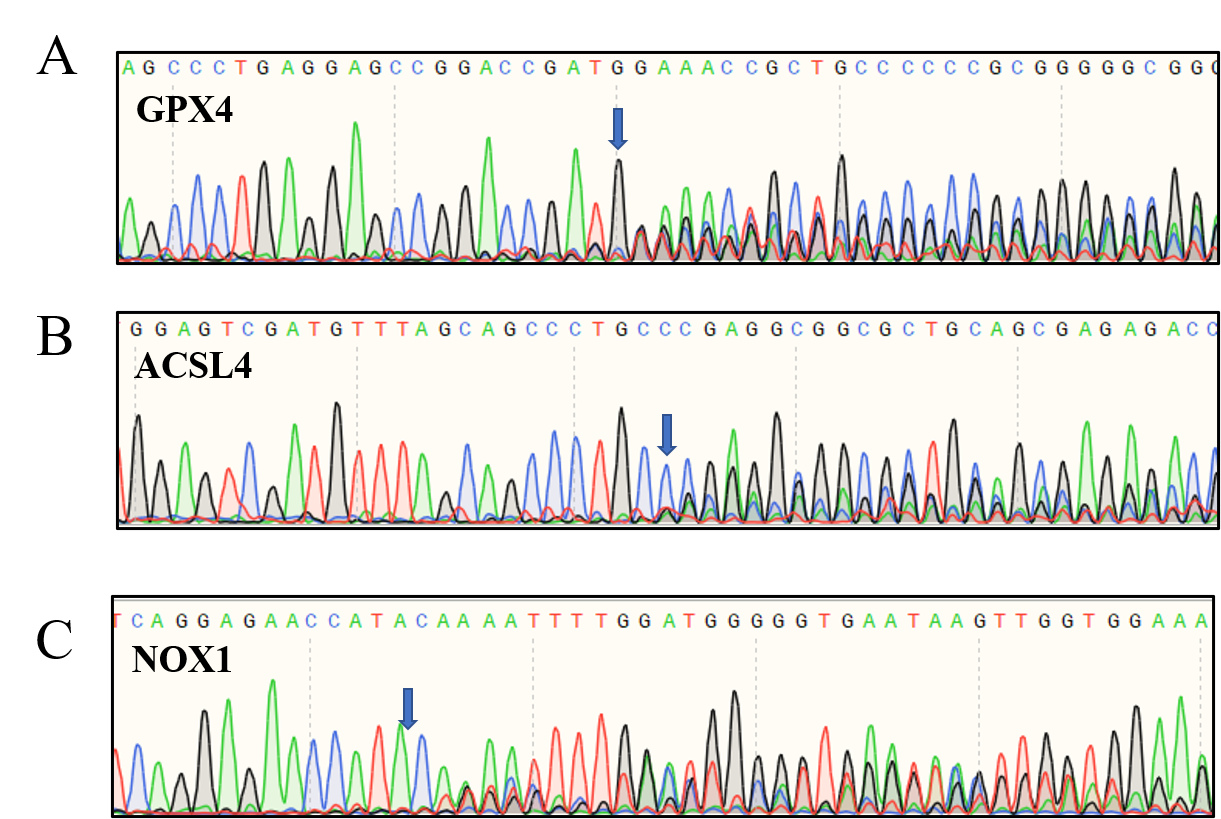


Supplementary Figure S5


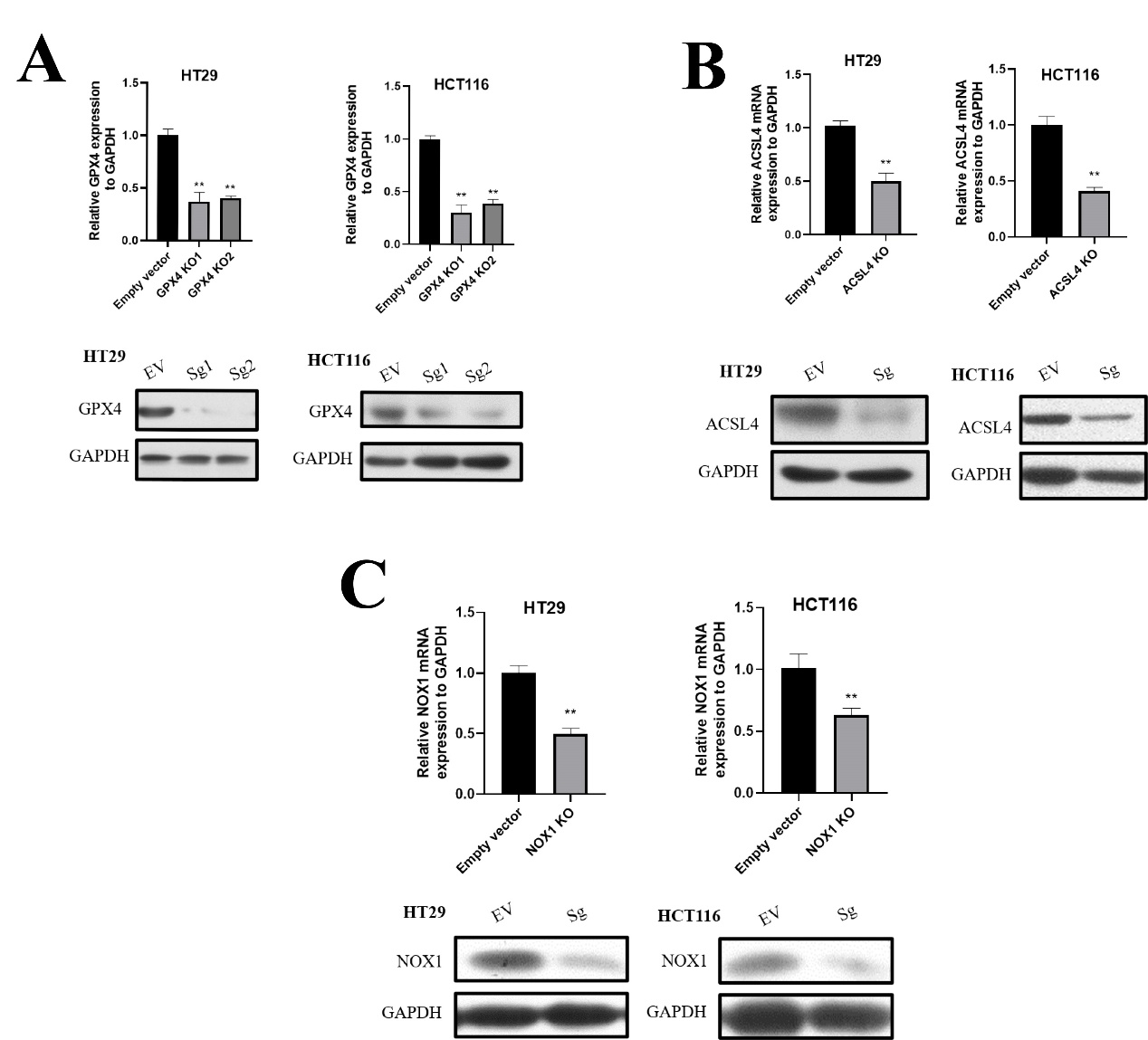


Supplementary Figure S6


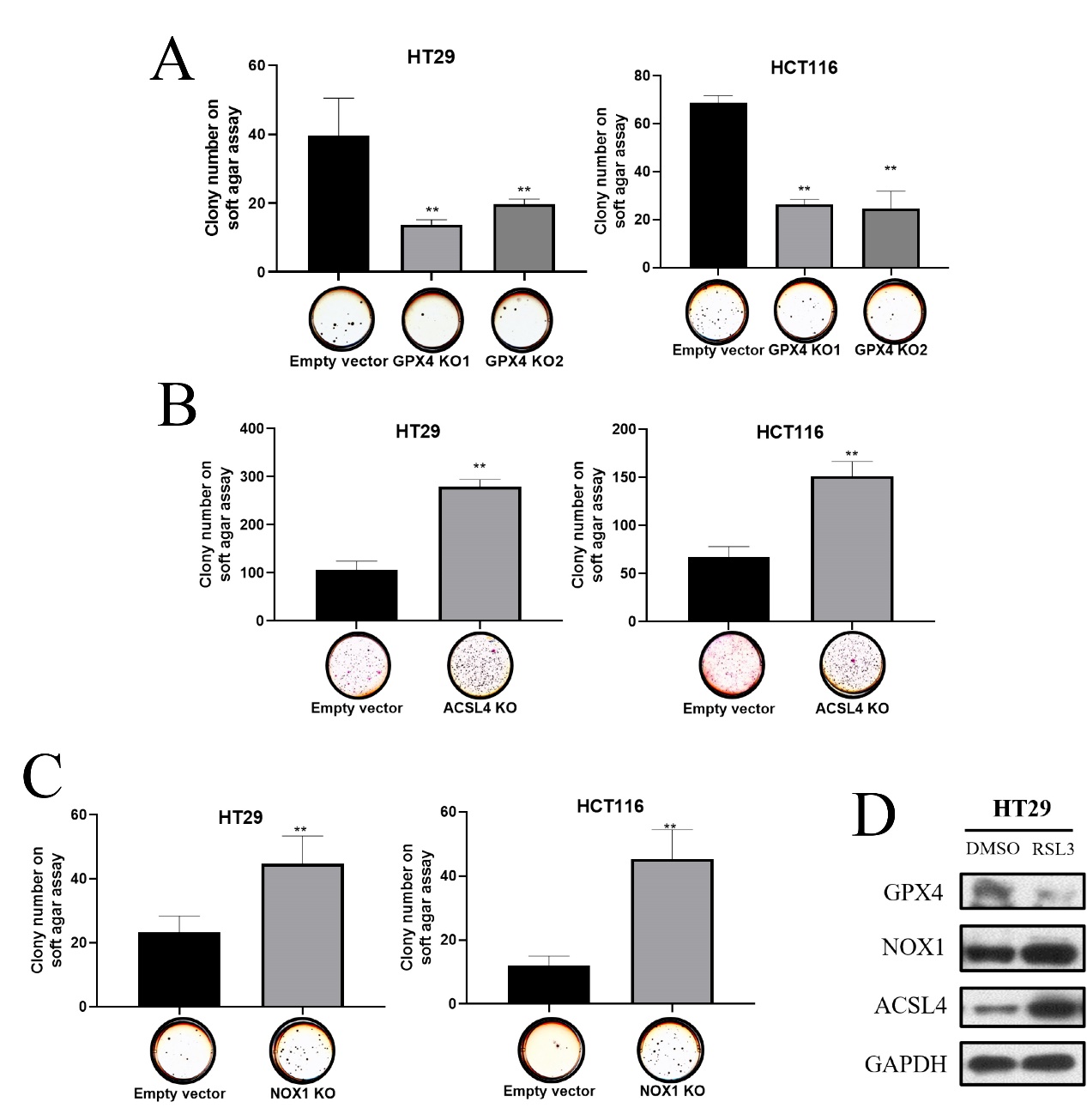


Supplementary Figure S7

Supplementary Figure S8

Supplementary Figure S9

Supplementary Figure S10

Supplementary Figure S11

Supplementary Figure S12

**Supplementary information**

Supplementary information 1

**Exploration of Ferroptosis-related genes in pubmed**

We searched the “pubmed” database for ferroptosis-related genes in cancer. The Keywords were “ferroptosis” OR “ferroptotic” AND “cancer” OR “tumor” OR “neoplasm”. The date was from 1. July. 1966 to 1. July.2020. The number of references was as follows:

| **Database** | **Key words** | **Number of reference** |
| --- | --- | --- |
| PubMed (1966-2020-7-1) | Ferroptosis OR ferroptotic | 1189 |
|  | Ferroptosis OR ferroptotic AND cancer OR tumor OR neoplasm | 623 |
|  | Ferroptosis OR ferroptotic AND cancer OR tumor OR neoplasm; article type: “article” OR “review” | 174 |

With the 174 publications, we further determined 42 ferroptosis related genes in Table S1.

Supporting information 2

**Tissue microarray construction, immunohistochemical staining**

1. **Tissue microarray construction**

As a retrospective study, all samples were formalin-fixed paraffin-embedded (FFPE) tissue blocks retrieved from Department of Pathology, Zhongshan Hospital, Fudan University. These FFPE tissue blocks were made for routine pathological examination after surgery, according to Chinese Standardization Criteria for Tumor Pathology Diagnosis (by National Health Commission of China). In this study, FFPE blocks of tumor tissues without necroses were selected as “Tumor samples”. The position of tissue blocks on the surgical specimen is shown as Figure 1. In Figure 1 was a surgical specimen of radical right hemicolectomy. Tumor region was marked by dotted line, and the necrotic region at tumor center was marked by solid line. FFPE tissue blocks sampled in the annular region between dotted and solid lines were selected as “Tumor samples”. Two pieces of “Tumor samples” were selected, located at different side of the tumor (in our Zhongshan Hospital, Fudan University, each tumor was required to sample more than 4 pieces at different side in this annular region for routine pathological examination, providing sufficient FFPE tissue blocks for selection). At the same time, normal mucosa tissues at colon resection margin were selected as “Normal mucosa sample”, as shown in Figure 1.

For tumor and normal specimen, tissue microarray (TMA) was constructed using FFPE tissue blocks as previously reported (PMID: 30988081). The diameter of tissue core was 2 mm. All samples were reviewed histologically by hematoxylin and eosin staining. Representative areas were marked on the paraffin blocks away from necrotic and hemorrhagic materials. Histological review was also constructed to avoid necrotic and hemorrhagic tumor regions.

For each patient, two cores of tumor tissues were taken from two tumor specimen as was shown in Figure 2(A). Tumor cores were taken in the middle of tumor sample piece, which were away from necrotic area. Correlated normal mucosa tissues were taken from two different areas of one “normal mucosa” in the middle of resection line (Figure 2B). Thus TMA was constructed (Figure 2C).


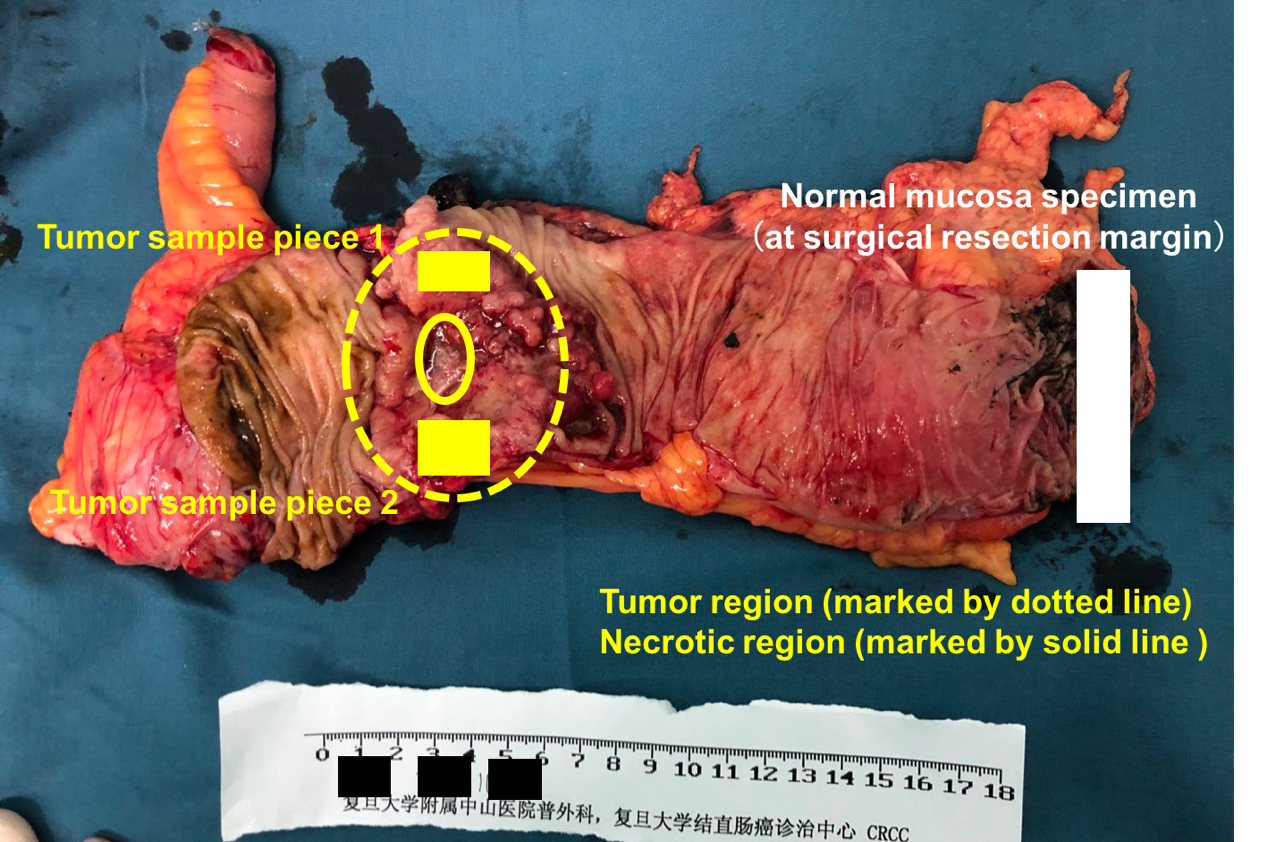


Figure 1: Position of the tissue blocks on surgical specimen


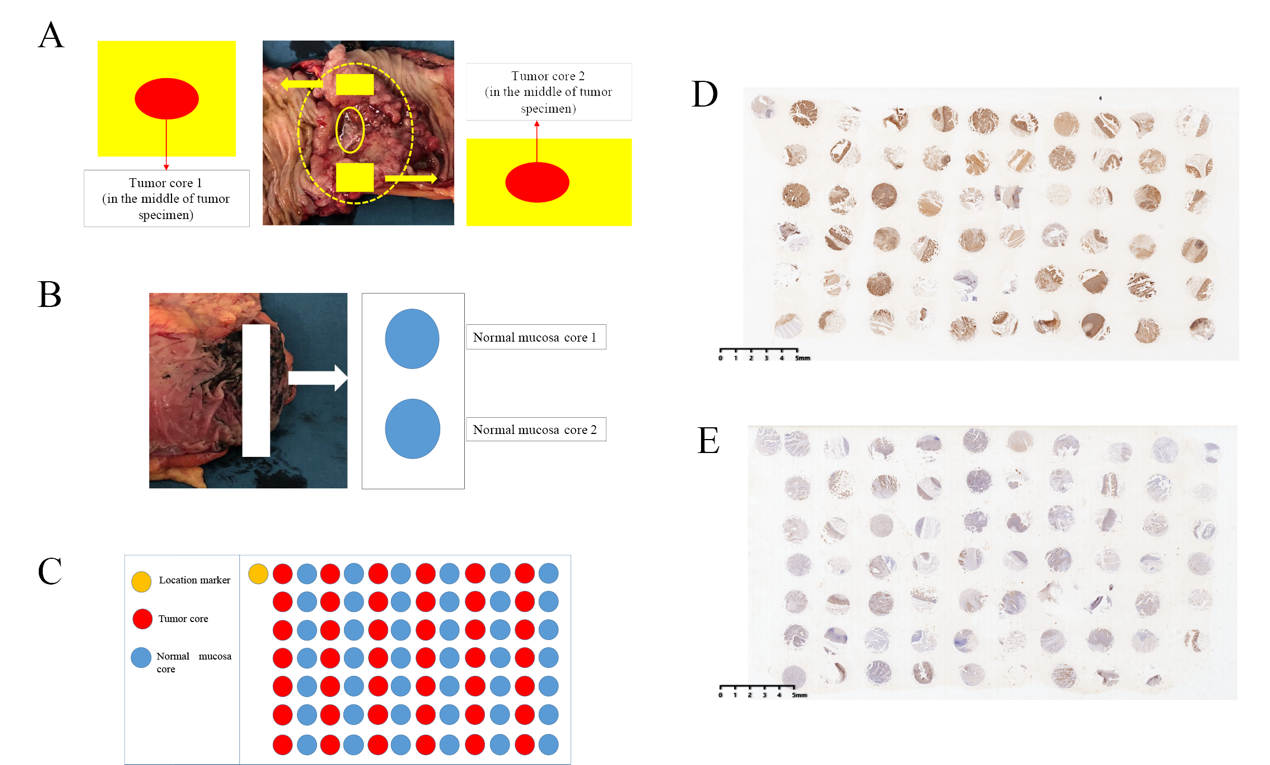


Figure 2: (A) Making tumor tissue cores; (B) Making normal mucosa tissue cores; (C) Tissue microarray of CRC cohort; (D) and (E) representative image of total immunohistochemical staining for tissue microarray.

1. **Immunohistochemical staining**

Briefly, sections were deparaffinized in xylene and graded alcohols, hydrated, and washed in phosphate-buffered saline. After the endogenous peroxidase was inhibited by 3% H_2_O_2_ for 30 minutes, the sections were heated in a pressure cooker for 5 minutes in unmasking solution (0.01 M sodium citrate buffer, pH = 6) and then incubated in 10% normal goat serum for 30 minutes. Primary antibodies were applied overnight in a moist chamber at 4°C. After the primary antibody was washed off, the EnVision Detection System (Peroxidase/DAB, Rabbit/Mouse, Dako) was used according to the instructions. Then the sections were counterstained with hematoxylin, dehydrated and mounted.

Supplementary information 3

**Evaluation of the stability and repeatability of the detection method for ferroptosis-related genes**

In our study, 2 pathologists, Dr Lingli Chen (Pathologist A) and Dr Jiamei Yao (Pathologist B) from Department of Pathology, Zhongshan Hospital, Fudan University performed the work of detecting ferroptosis-related markers, including the proportion of positive tumor cells in the tissues and the average intensity of positive tumor cells. For each patient, there were 2 tumor cores named as tumor core 1 and tumor core 2. And for each tumor core, 3 photos of HPF (200x) were taken at different area. A total of 2683 photos were taken for tumor cores of all patients. The IHC score from Pathologist A and Pathologist B were compared on each photo, using intraclass correlation coefficient (ICC) to evaluate the stability and repeatability of our detection method. According to the comparison between Pathologist A and B, in all photos of each tumor core, the R square were in high value > 0.95, showing good consistency. This confirmed the stability and repeatability of our detection method of IHC score. Details were as follows:

Figure 1: (A) GPX4 expression of all photos from Tumor Core 1 and Tumor Core 2 were compared from Pathologist A; (B) GPX4 expression of all photos from Tumor Core 1 and Tumor Core 2 were compared from Pathologist B; (C) GPX4 expression of all photos from Tumor Core 1 were compared between Pathologist A and B; (D) GPX4 expression of all photos from Tumor Core 2 were compared between Pathologist A and B. All these results demonstrated good consistency.

 Figure 2: (A) NOX1 expression of all photos from Tumor Core 1 and Tumor Core 2 were compared from Pathologist A; (B) NOX1 expression of all photos from Tumor Core 1 and Tumor Core 2 were compared from Pathologist B; (C) NOX1 expression of all photos from Tumor Core 1 were compared between Pathologist A and B; (D) NOX1 expression of all photos from Tumor Core 2 were compared between Pathologist A and B. All these results demonstrated good consistency.

 Figure 2: (A) FACL4 expression of all photos from Tumor Core 1 and Tumor Core 2 were compared from Pathologist A; (B) FACL4 expression of all photos from Tumor Core 1 and Tumor Core 2 were compared from Pathologist B; (C) FACL4 expression of all photos from Tumor Core 1 were compared between Pathologist A and B; (D) FACL4 expression of all photos from Tumor Core 2 were compared between Pathologist A and B. All these results demonstrated good consistency.

Supplementary information 4

**Comparison between normal sections and tissue microarray for immunohistochemical staining**

Immunohistochemical staining of Ferroptosis markers was compared between using normal sections and using tissue microarray. Intraclass correlation coefficient (ICC) was used to evaluate the consistency of normal sections and tissue microarray. ICC parameter selected as: two-way mixed model; absolute agreement; single measures. Details are as follows:

Figure 1: For GPX4 expression, IHC score detected using normal section versus tissue microarray

Figure 2: For NOX1 expression, IHC score detected using normal section versus tissue microarray

Figure 3: For FACL4 expression, IHC score detected using normal section versus tissue microarray
